# Heavy‐Atom Tunneling in Semibullvalenes: How Driving Force, Substituents, and Environment Influence the Tunneling Rates

**DOI:** 10.1002/chem.202001202

**Published:** 2020-07-28

**Authors:** Tim Schleif, Jörg Tatchen, Julien F. Rowen, Frederike Beyer, Elsa Sanchez‐Garcia, Wolfram Sander

**Affiliations:** ^1^ Lehrstuhl für Organische Chemie II Ruhr-Universität Bochum 47780 Bochum Germany; ^2^ Computational Biochemistry Universität Duisburg-Essen 45117 Essen Germany

**Keywords:** confinement, heavy-atom tunneling, matrix isolation, SCT calculations, solvent effects

## Abstract

The Cope rearrangement of selectively deuterated isotopomers of 1,5‐dimethylsemibullvalene **2 a** and 3,7‐dicyano‐1,5‐dimethylsemibullvalene **2 b** were studied in cryogenic matrices. In both semibullvalenes the Cope rearrangement is governed by heavy‐atom tunneling. The driving force for the rearrangements is the small difference in the zero‐point vibrational energies of the isotopomers. To evaluate the effect of the driving force on the tunneling probability in **2 a** and **2 b**, two different pairs of isotopomers were studied for each of the semibullvalenes. The reaction rates for the rearrangement of **2 b** in cryogenic matrices were found to be smaller than the ones of **2 a** under similar conditions, whereas differences in the driving force do not influence the rates. Small curvature tunneling (SCT) calculations suggest that the reduced tunneling rate of **2 b** compared to that of **2 a** results from a change in the shape of the potential energy barrier. The tunneling probability of the semibullvalenes strongly depends on the matrix environment; however, for **2 a** in a qualitatively different way than for **2 b**.

## Graphical abstract

**The influence of cryosolvation** and confinement on the heavy‐atom tunneling process in the Cope rearrangement of selectively deuterated dicyanosemibullvalene derivatives has been studied. The experimentally determined reaction rates nicely agree with TST+SCT calculations and provide insights into the compensation of barrier heights and widths as determining factors of tunneling processes (see figure).

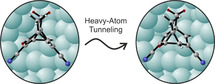

## Introduction

**Scheme 1:**
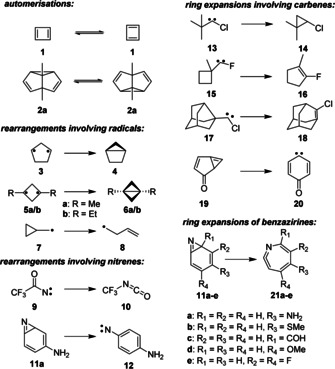
Previously reported reactions with experimental evidence for heavy‐atom tunneling: Automerisations of cyclobutadiene **1** 6 and 1,5‐dimethylsemibullvalene **2 a** 4 (see also the conformational tunneling of *gauche*‐butadiene),^7^ ring closures of cyclopentane‐1,3‐diyl **3** 8 and cyclobutane‐1,3‐diyls **5**,^9^ ring opening of cyclopropyl carbinyl **7**,3b rearrangement of trifluoroacetyl nitrene **9**,^10^ ring opening of benzazirine **11 a**,2c ring expansions of *tert*‐butylchlorocarbene **13**,^11^ fluorocarbene **15**,^12^ noradamantylchlorocarbene **17**,^13^ oxocyclohexadienylidene **19** 14 and benzazirines **11 a**–**e**.^2^

Quantum mechanical tunneling describes the penetration of potential energy barriers without crossing the barriers, and thus provides alternative routes to classical thermal reaction pathways. The probability for tunneling reactions is highly dependent on the width and height of the barrier as well as on the mass of the tunneling system. The latter dependency explains why tunneling processes dominated by the movement of heavier atoms like carbon, often termed „heavy‐atom tunneling“, have only rarely been observed experimentally (Scheme 1) compared to a plethora of well‐documented examples for hydrogen tunneling.^1^ Though the instances of heavy‐atom tunneling reported so far cover a broad range of reactions, the formation or opening of three‐membered rings is a common motif in many of these reactions. This results from the minimal structural changes and thus narrow barriers in many of these processes. The ring expansion of benzazirines is one of the few examples where the tunneling rates as a function of the shape of the barrier were studied in some detail.^2^

**Scheme 2:**
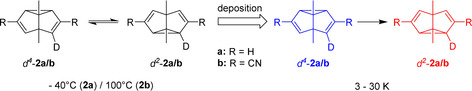
Representation of the experimental approach to study heavy‐atom tunneling in the Cope rearrangement of semibullvalenes.

While most of the reactions dominated by heavy‐atom tunneling were chance discoveries, small curvature tunneling (SCT) calculations have some predictive power^3^ as demonstrated by our experimental investigation of the Cope rearrangement of 1,5‐dimethylsemibullvalene **2 a**.^4^ Based on a computational study by Borden et al.,^5^ selective deuteration in 2‐/4‐position was chosen to lift the degeneracy of this Cope rearrangement (Scheme 2). The two resulting isotopomers slightly differ in their zero‐point vibrational energies (ZPVE) due to changes in the C−H bending frequencies. While the isotopic label barely affects the thermodynamic equilibrium at elevated temperatures, it results in a significant thermodynamic driving force at cryogenic temperatures, favoring the more stable isotopomer *d* ^*2*^‐**2 a**. Thus, rapid quenching of the room temperature equilibrium by deposition onto a cold spectroscopic window at 3–30 K allowed us to measure the reaction rates at these temperatures. The rearrangement is observed at lowest temperatures despite the absence of sufficient thermal energy to surmount the activation barrier, and between 3 and 30 K the rates are nearly independent of temperature. This strongly indicates that under these conditions the rearrangement is governed by heavy‐atom tunneling.

Herein, we report how the driving force, substituents, and the matrix environment influence the tunneling rates of the Cope rearrangements of semibullvalenes.

## Results and Discussion

### Influence of substituents

The experimental approach restricts the choice of suitable target molecules to symmetrically substituted, deuterated semibullvalenes. A suitable molecule is the 3,7‐dinitrile derivative **2 b** which is easier to synthesize and handle than the parent semibullvalene **22** while it is predicted to show an activation barrier only slightly higher than that of **2 a** (Table 1).

**Table 1:** DFT calculated^[a]^ energy and geometric parameters of **2 a**, **2 b**, and the parent semibullvalene **22**.

Property	**2 a**		**2 b**		**22**	
	calcd.	exptl.	calcd.	exptl.	calcd.	exptl.
Δ*H* ^≠^ (*E*_A_) [kcal mol^−1^]	3.1 (3.2)	4.5 (4.8)^15^	4.8 (4.9)	5.6^16^	4.0 (4.0)	4.8 (5.1)^17^
ΔΔ*C*–*C* ^[b]^ [Å]	0.72	–	0.72	0.21^18^	0.75	0.66^19^
ΔZPVE(*d_1_*)^[c]^ [kcal mol^−1^]	−0.08	−0.12^20^	−0.08	−0.08^21^	−0.08	−0.07^22^

[a] All calculations were performed at the B3LYP/6–311G(d,p) level of theory. [b] ΔΔ*C*–*C* are the differences in the distances between the carbon atoms C2/C8 and C4/C6, respectively. [c] ΔZPVE are the differences in zero‐point vibrational energies between the corresponding *d_1_*‐isotopomers.

**Figure 1:**
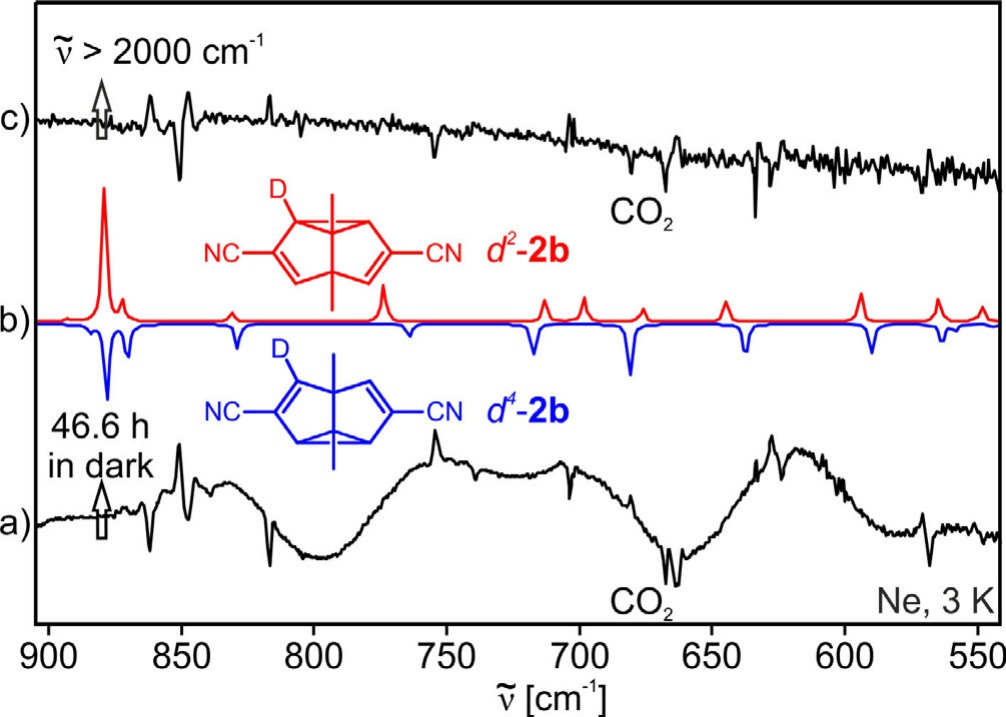
IR difference spectra showing the Cope rearrangement of *d* ^*4*^‐**2 b** and its photochemical reversion. a) Experimental IR difference spectrum obtained after keeping a neon matrix containing a mixture of *d* ^*2*^‐**2 b** and *d* ^*4*^‐**2 b** in the dark at 3 K for 46.6 h. b) Theoretical IR spectra of *d* ^*4*^‐**2 b** (pointing downwards) and *d* ^*2*^‐**2 b** (pointing upwards) calculated at the B3LYP/6–311G(d,p) level of theory. c) Experimental IR difference spectrum obtained after subsequent broadband irradiation ($\tilde{\nu }$ >2000 cm^−1^) of this matrix with the IR Globar for 4.5 hours at 3 K. The peak at approx. 670 cm^−1^ pointing downwards in either of the experimental IR difference spectra is due to CO_2_.

The room temperature equilibrium mixture of the isotopomers *d* ^*2*^‐**2 b** and *d* ^*4*^‐**2 b** (containing non‐deuterated **2 b** as minor impurity) was deposited with a large excess of neon on a CsI window at 3 K. The IR spectra of the isotopomers noticeably differ in the mid IR region which allows to quantify changes in the concentrations of *d* ^*2*^‐**2 b** and *d* ^*4*^‐**2 b**. Keeping the matrix in the dark for approximately two days results in a decrease of the IR signals of the less stable isotopomer *d* ^*4*^‐**2 b** and concomitant increase of the *d* ^*2*^‐**2 b** signals (Figure 1). This is in accordance with our findings for *d_1_*‐**2 a**.^4^ With approx. 5 kcal mol^−1^, the Cope rearrangement of **2 b** shows an even higher activation barrier than that of **2 a**; the observation of this rearrangement at cryogenic temperatures thus strongly indicates heavy‐atom tunneling. As expected, reference experiments with non‐deuterated **2 b** did not result in any time‐dependent changes in the IR spectra.

IR broadband irradiation above 2000 cm^−1^ results in an approximately 1:1 ratio of *d* ^*2*^‐**2 b** and *d* ^*4*^‐**2 b**, and therefore appropriate IR band‐pass filters had to be used to suppress any IR induced rearrangement that could compete with the tunneling process. However, after the kinetics measurements, IR induced rearrangement was used to increase the concentration of the less stable isotopomer *d* ^*4*^‐**2 b** for subsequent kinetics runs using the same matrix.

The kinetics of the Cope rearrangement *d* ^*4*^‐**2 b**→*d* ^*2*^‐**2 b** was followed by monitoring changes in the intensities of the characteristic IR peaks at 755 cm^−1^ assigned to *d* ^*2*^‐**2 b** and at 817 cm^−1^ assigned to *d* ^*4*^‐**2 b**. These peaks do not significantly overlap and show sufficiently high intensity for a quantitative assessment. The kinetics of the rearrangement were found to be highly dispersive, as also observed for **2 a**,^4^ necessitating the use of the stretched exponential approach by Wildman and Siebrand [Eq. (1)].^23^ In this approximation, a dispersion coefficient β is employed to account for the presence of a variety of non‐uniform matrix sites exhibiting individual, slightly varying rate constants. The inclusion of a constant offset c into the expression allows for the simultaneous fit of the increasing as well as decreasing IR intensities.

(1) $$I=I_{0}•e^{{-\left(kt\right)}^{\beta }}+c\;with\;0<\beta <1$$

The evaluation of the kinetics data reveals that changing the matrix temperature does not influence the rates significantly (Table 2). With an experimental activation enthalpy of 5.6 kcal mol^−1^,^16^ the eightfold increase in temperature in N_2_ should result in a rate acceleration of approx. 10^357^ assuming a conventional thermal reaction, contrary to the observations.

**Table 2:** Rate constants^[a]^ apparent half‐life for the Cope rearrangements of semibullvalenes *d_1_*‐**2 a** and *d_1_*‐**2 b** in various matrices.

*T* [K]	Matrix	*d* ^*4*^‐**2 b**→*d* ^*2*^‐**2 b**		*d* ^*4*^‐**2 a**→*d* ^*2*^‐**2 a** 4	
		*k* **⋅**10^−5^ [s^−1^]	*τ*_app_ [h]	*k* **⋅**10^−5^ [s^−1^]	*τ*_app_ [h]
3	N_2_	5.6±0.8	3.0	2.5±0.1	0.7
8	N_2_	4.7±0.9	3.5		
13	N_2_	11.2±2.6	1.5		
18	N_2_	6.4±0.9	2.6		
23	N_2_	6.3±2.0	2.6		
3	Ar	6.0±1.3	2.7	1.5±0.1	1.2
25	Ar	9.8±2.9	1.7	4.8±0.5	0.4
3	*p‐*H_2_	not observed		1.1±0.1	1.7
3	Xe	2.6±0.9	6.3	not observed	
35	Xe	3.9±0.9	4.2		
3	neat	not observed		not observed	
3	Ne	2.8±0.2^[b]^	6.8	1.7±0.1	1.3
6	Ne	2.8±0.4^[b]^	7.0

[a] Rate constants fitted to Eq. (1) with *β*=0.8 for **2 a** in Ne, 1.0 for **2 b** in Ne and *β*=0.7 for **2 b** in all other matrices. [b] Averaged over two different experiments within the same matrix.

**Figure 2:**
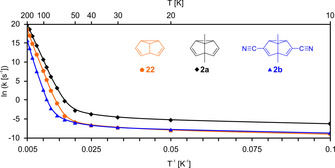
Calculated (SCT+TST, B3LYP‐D3/6‐31G(d)) Arrhenius plots for semibullvalene **22**, 1,5‐dimethylsemibullvalene **2 a**, and 3,7‐dicyano‐1,5‐dimethylsemibullvalene **2 b**.

In solid Ar, N_2_, or Ne the Cope rearrangement of *d* ^*4*^‐**2 b** is noticeably slower than that of *d* ^*4*^‐**2 a** (by a factor of approx. 5). Rate calculations for **2 a**, **2 b**, and **22** using SCT+TST theory reveal that, as expected, the barrier height determines the rearrangement rates in the high‐temperature regime: *k*_**2 a**_>*k*_**22**_>*k*_**2 b**_ (Figure 2). However, at cryogenic temperatures, in the tunneling regime, **2 b** and **22** are predicted to exhibit almost identical reaction rates: *k*_**2 a**_>*k*_**22**_≈*k*_**2 b**_.

According to the Wentzel–Kramers–Brillouin approximation,^24^ probabilities for one‐dimensional tunneling through a parabolic barrier only depend on three parameters [Eq. (2)]:

(2) $$P\left(E\right)=e^{-\pi^{2}w\sqrt{2m{(V}_{0}-E)}/h}$$

The effective tunneling mass *m* of the three semibullvalenes is identical^5^ and the activation energy *E_A_* (as an approximate measure for *V_0_*−*E*)^25^ could already be ruled out as the cause of the increased tunneling rate of **2 b**. Thus, we conclude that **2 b** exhibits a noticeably different barrier shape with a smaller tunneling width *w* to compensate for the increased barrier height. To confirm this hypothesis, the energy profiles for the Cope rearrangement of several semibullvalenes were compared. Besides **22**, **2 a**, and **2 b**, the two additional reference compounds **2 c** and **2 d** were investigated in order to shed light on the electronic influence of the nitrile groups in **2 b**. The fluoro substituents in **2 c** mimic the electron‐withdrawing properties of their pseudohalogen analogues, whereas the acetylene moieties in **2 d** are isoelectronic to −C≡N and conserve their triple bond motif.

**Figure 3:**
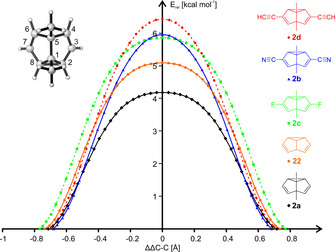
Potential energy scan of semibullvalenes regarding to the difference in the distances between the carbon atoms C2 and C8 and between C4 and C6 (ΔΔ*C*–*C*). Calculations at the B3LYP/6‐311G(d,p) level of theory; for the equivalent plot of the reaction paths used for the SCT calculations see Supporting Information.

**Figure 4:**
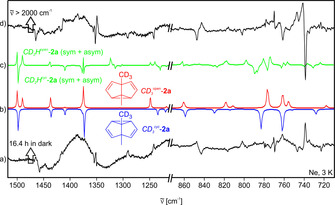
IR spectra showing the Cope rearrangements of *CD_3_* ^*cyc*^‐**2 a** and *CD_2_H* ^*cyc*^‐**2 a**. a) Experimental IR difference spectrum obtained after keeping a neon matrix containing a mixture of *CD_3_* ^*cyc*^‐**2 a** and *CD_3_* ^*open*^‐**2 a** (with possible contaminations by *CD_2_H* ^*cyc*^‐**2 a** and *CD_2_H* ^*open*^‐**2 a**) in the dark for 16.4 hours at 3 K. b) Calculated IR spectra of *CD_3_* ^*cyc*^‐**2 a** (pointing downwards) and *CD_3_* ^*open*^‐**2 a** (pointing upwards), B3LYP/6–311G(d,p). c) Calculated difference spectrum of a combination of the two degenerate asymmetric and the symmetric rotamers of *CD_2_H* ^*cyc*^‐**2 a** (pointing downwards) and the corresponding rotamers of *CD_2_H* ^*open*^‐**2 a** (pointing upwards), B3LYP/6–311G(d,p). d) Experimental IR difference spectrum obtained after subsequent broadband irradiation ($\tilde{\nu }$ >2000 cm^−1^) of this matrix at 3 K.

As evident from the reaction coordinates for the Cope rearrangements of **2 a**–**d** and **22** in Figure 3, two distinct barrier shapes can be identified: 1,5‐dimethylsemibullvalene **2 a** possesses a similar barrier shape than the parent semibullvalene **22**, both exhibiting a full width at half height (FWHH) of (0.43±0.01) Å despite different barrier heights (Table 3). In marked contrast to this, the widths of the barriers for the Cope rearrangement of **2 b**, the difluoride **2 c** or 3,7‐diacetylene **2 d** noticeably differ despite being very similar in barrier height. The impact of these differences in the barrier widths on the tunneling probabilities can be quantified via Eq. (2), with the values for FWHH from the energy profiles in Figure 4 being used as an approximate measure^25^ of the barrier width *w* and *E_A_* as a measure for *V_0_*−*E*. An analogous analysis has been demonstrated to provide quantitatively correct predictions for the rates of the ring expansions of benzazirines **11 d/e**.2d Likewise, the relative tunneling probabilities as obtained from Eq. (2) of all semibullvalenes nicely correlate with both the rate constants gained from experiments as well as the ones from SCT calculations (Table 3).

**Table 3:** Tunneling probabilities and rate constants^[a]^ of semibullvalenes **2 a**–**d** and **22** as function of barrier height (*E*_A_) and width (FWHH).

Species	*E*_A_ [kcal mol^−1^]	FWHH [Å]	*P*_rel_	$k_{SCT+TST}^{10K}$ [s^−1^]	$k_{Ne}^{3K}\;[$ s^−1^]
**22**	4.0	0.42	7×10^−2^	1.4×10^−4^	/
**2 a**	3.2	0.43	1	1.8×10^−3^	1.7×10^−4 [4]^
**2 b**	4.9	0.37	2×10^−1^	1.5×10^−4^	2.8×10^−5^
**2 c**	4.6	0.50	2×10^−5^	1.7×10^−8^	/
**2 d**	5.3	0.40	3×10^−3^	4.5×10^−6^	/

[a] Rate constants from SCT calculations (*k*_SCT+TST_) compared to experimental rate constants in neon at 3 K (*k*_Ne_).

The underlying cause for the narrower barrier for the Cope rearrangement of **2 b** (with respect to the barrier height) has already been indirectly stated by Dannenberg et al.^26^ in their MNDO study of the Cope rearrangement of isomeric dicyanosemibullvalenes. While the two cyano groups in 3‐ and 7‐position stabilize the ground states due to conjugation, they barely affect the homo‐conjugated transition state with the highest occupied molecular orbital having a node at carbons C3 and C7. This hypothesis not only explains the greater height of the activation barrier, but also elucidates the rapid stabilization upon increasing the bond localization between the carbons C3 (C7) and C2 (C8) or C3 (C7) and C4 (C6), respectively. The same mechanism is at work in **2 d** which also experiences a rather steep drop in energy upon smaller changes in ΔΔ*C*–*C* due to the onset of conjugation. Subsequently, **2 d** shows a narrow barrier comparable in shape to **2 b**, although the greater height results in a smaller tunneling probability.

### Influence of the environment

The order of the rate constants at 3 K in various matrices noticeably differs between **2 a** (*p*‐H_2_<Ne≈Ar<N_2_) and **2 b** (Xe≈Ne<N_2_≈Ar), with no clear trend with respect to macroscopic properties of these matrices (e.g. polarizability or melting point). Remarkable is the quenching of the tunneling rearrangements in the neat compounds (deposition at 3 K without matrix) and in either solid Xe (**2 a**) or *p*‐H_2_ (**2 b**). For *p*‐H_2_ it was reported that traces of *o*‐H_2_ (<0.1 %) cluster around polar dopants which results in the quenching of tunneling processes (e.g. in acetylacetone or 2‐chloromalonaldehyde).^27^ Since the dinitrile **2 b** is calculated to be highly polar (*μ*=6.7 D from B3LYP/6–311G(d,p)) and exhibits rather broad IR bands when isolated in *p*‐H_2_, a similar phenomenon might also inhibit its heavy‐atom tunneling, though the underlying mechanism is not understood.

It was found that polar and polarizable solvents can significantly lower the barrier for the rearrangement of semibullvalenes due to the greater polarizability of the homo‐conjugated transition states.^28^ Moreover, some semibullvalene derivatives have been reported to exhibit distorted geometries in the solid state (as also evident from the unusually small ΔΔ*C*–*C* for **2 b** in Table 1),^29^ which can lead to localized structures in condensed phase despite having a delocalized homoaromatic ground state in the vapor phase.^30^ Thus, the Cope rearrangement of semibullvalenes is governed by a complex interplay of confinement and solvation. Since this is highly specific for individual semibullvalenes, these subtle effects might lead to the contrasting behavior of **2 a** and **2 b** in solid *p*‐H_2_ and Xe.

A common feature of the Cope rearrangement of **2 a** and **2 b** is that even during prolonged experiments (up to several days) the thermodynamic equilibrium between the two isotopomers is not reached. After more than ten half‐life times the experimental isotopomeric ratios *d* ^*2*^‐**2 b** : *d* ^*4*^‐**2 b** are largely differing from the ratios expected from the (experimentally determined)^21^ difference in ZPVE of −0.08 kcal mol^−1^ (see Supporting Information). The isotopomeric ratios obtained in various matrices show no obvious correlation with the matrix temperature or the type of matrix and generally reach a final value *d* ^*2*^‐**2 b** : *d* ^*4*^‐**2 b** of roughly 2.

This observation is in line with our earlier findings for **2 a**, but also with similar reports by Nakata et al. on the tunneling of hydroquinones:^31^ They found that these compounds exhibit ratios far different from the thermodynamic equilibrium at cryogenic temperatures and hypothesized that the inhomogeneous matrix environment subtly varies the energy differences between conformers. This hypothesis might also explain why the hydrogen‐tunneling of malondialdehyde, observable in gas phase, is inhibited in a rare gas matrix^32^ or the tunneling splitting is reduced in spectra of tropolone in neon matrices compared to the gas phase.^33^ In each of these cases the asymmetry of the PES induced by the rigid environment might affect the tunneling processes. Such phenomenon has even been explicitly predicted by Bredtmann et al. for the Cope rearrangement of semibullvalene within small external electric fields^34^ as they might be experienced in crystalline environments.

### Influence of the thermodynamic driving force

**Scheme 3:**
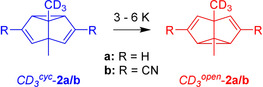
Cope rearrangements of *CD_3_*‐**2 a/b**.

In addition to the deuterium label in 2‐ or 4‐position, the selective deuteration of one of the methyl groups was chosen as an alternative means to lift the degeneracy of the Cope rearrangement of **2 a/b** (Scheme 3). Askani et al. demonstrated that the isotopomers of *CD_3_*‐**2 a** differ in ZPVE by approx. 0.01 kcal mol^−1^,^35^ nearly one order of magnitude less than the corresponding isotopomers of *d_1_*‐**2 a**, which barely depends on the degree of deuteration (for an extended discussion, see Supporting Information) or symmetry (Table 4).

**Table 4:** Differences in the zero‐point vibrational energies (ΔZPVE) for several isotopologues of **2 a/b**.

Isotopologue		**2 a**		**2 b**
		calcd.^[a]^ ΔZPVE [cal mol^−1^]	exptl.^[b]^ Δ*H* ^0^ [cal mol^−1^]	calcd.^[a]^ ΔZPVE [cal mol^−1^]
CD_3_		−19.5	−9.6	−6.3
CD_2_H	sym^[c]^	−16.9	−6.5	−8.1
	asym^[c]^	−11.3		−2.5
CDH_2_	sym^[c]^	−3.1	could not be resolved	+2.5
	asym^[c]^	−9.4		−5.0
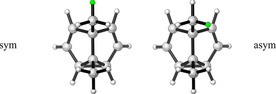				

[a] Calculated at the B3LYP/6‐311G(d,p) level of theory. [b] Experimental values, ref. [46]. [c] Symmetrical (sym) or asymmetrical (asym) with respect to the C_s_ point group of **2 a/2 b**. For clarification, see Figure with atoms highlighted in green marking the position of the protium in the *CD_2_H* group or of the deuterium in the *CDH_2_* group, respectively.

*CD_3_*‐**2 a** was deposited in a neon matrix at 3 K, and changes in the IR spectrum were monitored over time. These changes indicate a rearrangement of *CD_3_* ^*cyc*^‐**2 a** to the slightly more stable *CD_3_* ^*open*^‐**2 a** (Figure 4). Contributions of the rearrangement of the partially deuterated isotopologue *CD_2_H*‐**2 a** could not be excluded, as additional features in the region around 1300 cm^−1^ might result from C−H scissoring motions only present in *CD_2_H*‐groups.

**Figure 5:**
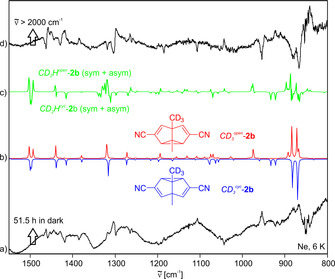
IR spectra showing the Cope rearrangement of *CD_3_* ^*cyc*^‐**2 b**. a) Experimental IR difference spectrum obtained after keeping a neon matrix containing a mixture of *CD_3_* ^*cyc*^‐**2 b** and *CD_3_* ^*open*^‐**2 b** (with possible contaminations by *CD_2_H* ^*cyc*^‐**2 b** and *CD_2_H* ^*open*^‐**2 b**) in the dark for 51.5 hours at 6 K. b) Calculated IR spectra of *CD_3_* ^*cyc*^‐**2 b** (pointing downwards) and *CD_3_* ^*open*^‐**2 b** (pointing upwards), B3LYP/6–311G(d,p). c) Calculated IR spectra of a combination of the two degenerate asymmetric and the symmetric rotamers of *CD_2_H* ^*cyc*^‐**2 b** (pointing downwards) and the corresponding rotamers of *CD_2_H* ^*open*^‐**2 b** (pointing upwards), B3LYP/6–311G(d,p). d) Experimental IR difference spectrum obtained after subsequent broadband irradiation ($\tilde{\nu }$ >2000 cm^−1^) of this matrix at 6 K.

An analogous procedure with *CD_3_*‐**2 b** gave similar results: The IR difference spectrum resulting from keeping a neon matrix containing the sample in the dark for 52 hours agrees well with the expected rearrangement *CD_3_* ^*cyc*^‐**2 b**→*CD_3_* ^*open*^‐**2 b**. Nonetheless, as with *CD_3_*‐**2 a**, contributions from *CD_2_H*‐**2 b** might explain the peak structure around 1300 cm^−1^ (Figure 5).

The rate constants for the rearrangements of *CD_3_*‐**2 a** strongly resemble that for *d_1_*‐**2 a** in neon at 3 K, although the discrepancies in β prevent a more extensive mechanistic interpretation (Table 5). The rate constant for the rearrangement of *CD_3_*‐**2 b** also agrees reasonably well with the rate constant for *d_1_*‐**2 b**. Strikingly, the final isotopomer ratios also do not vary from the values found for their monodeuterated counterparts, despite significant differences in ZPVE and thus predicted Boltzmann distributions (see Supporting Information). This result casts doubt on the hypothesis that the deviation from the thermodynamic equilibrium is (solely) caused by the environment distorting the PES, although we do not have alternative explanations.

**Table 5:** Rate constants from Equation (1) with β as parameter^[a]^ for the Cope rearrangements of semibullvalenes **2 a** 4 and **2 b** measured in neon matrices at different temperatures.

*T* [K]	*CD_3_* ^*cyc*^‐**2 a**→*CD_3_* ^*open*^‐**2 a**		*CD_3_* ^*cyc*^‐**2 b**→*CD_3_* ^*open*^‐**2 b**	
	*k* **⋅**10^−4^ [s^−1^]	*τ*_app_ [h]	*k* **⋅**10^−5^ [s^−1^]	*τ*_app_ [h]
3	2.2±0.3^[b]^	0.7	5.5±0.5^[b]^	1.8
6	1.1±0.2^[b]^	1.4	7.2±0.7^[b]^	1.4
		
	*d* ^*4*^‐**2 a**→*d* ^*2*^‐**2 a**		*d* ^*4*^‐**2 b**→*d* ^*2*^‐**2 b**	
3	1.7±0.1	1.3	2.8±0.2^[b]^	6.8

[a] *β*=0.8 (for **2 a**)/1.0 (for **2 b**)/0.65 (for *CD_3_*‐**2 a**)/0.35 (for *CD_3_*‐**2 b**). [b] Averaged over two different experiments within the same matrix.

## Conclusions

10 years ago, Borden et al. proposed an experiment that would allow to test their prediction that semibullvalene undergoes a rapid degenerate Cope rearrangement via quantum chemical tunneling at cryogenic temperatures.^5^ The basis of this experiment is to use asymmetric isotopic labelling and to use the equilibrium isotope effect to shift the equilibrium to one of the isotopomers. While we could verify this prediction for 1,5‐dimethylsemibullvalene **2 a**,^4^ important questions remained open: (i) Does the energy difference between the isotopomers (the thermodynamic driving force) influence the tunneling rates? (ii) How sensitive are the tunneling rates on environmental effects in different matrices? (iii) Do these effects (thermodynamics and environment) show the same trends in different derivatives of semibullvalene?

In order to answer question (i) we studied the isotopologue *CD_3_*‐**2 a** with one of the methyl groups deuterated. Although the difference in zero‐point vibrational energy between the two isotopomers of *CD_3_*‐**2 a** is only roughly 10 % of that between *d* ^*2*^‐**2 a** and *d* ^*4*^‐**2 a**, the tunneling rates are the same within error limits. The same was observed for the corresponding isotopologues of the dicyano semibullvalene **2 b**. This indicates that the tunneling rates are independent of the thermodynamic driving force. But not only the tunneling rates are the same but also the final ratios of the isotopomers. The tunneling rearrangement never reaches the expected thermodynamic equilibrium and the final ratios are independent of the thermodynamic driving force. This indicates that the isotopomer ratios are governed by kinetics, not thermodynamics.

We already showed that the kinetics of the tunneling rearrangement of **2 a** are very dependent on the matrix,^4^ and for **2 b** we find a similar behavior. Notably, tunneling is only observed for matrix‐isolated semibullvalenes **2 a** and **2 b**, whereas at the same temperatures the neat, microcrystalline compounds do not show any rearrangement. Interestingly, **2 a** shows tunneling in *p*‐H_2_ but not in xenon, whereas **2 b** undergoes tunneling in xenon but not in *p*‐H_2_. Thus, there is no obvious answer for question (iii). While it is known that the transition‐state energies of the Cope rearrangement of semibullvalenes depend on a subtle interplay between confinement and solvation,^28^, ^29^, ^30^ there is no obvious explanation for the observed difference in matrix effects between **2 a** and **2 b**. Even the weakly interacting matrices Ne and *p*‐H_2_ show pronounced matrix effects, and the magnitude of these effects is different in **2 a** and **2 b** (Table 2). Gas phase data cannot be measured but results from SCT+TST calculations are reliable enough to compare derivatives of semibullvalenes. An interesting finding is that, despite the dicyanosemibullvalene **2 b** having a considerably larger activation barrier than **2 a**, the tunneling rates for both are predicted to be almost identical. This results from the barrier width strongly influencing the tunneling rates while not affecting the thermal rearrangement. The influence of cryosolvents on heavy‐atom tunneling kinetics has rarely been studied, and therefore the data presented here provide an important guide for future studies to untangle the general principles behind the complex influences of confinement and solvation on tunneling.

## Experimental Section

### Synthesis

1,5‐dimethylsemibullvalene **2 a** and 3,7‐dicyano‐1,5‐dimethylsemibullvalene **2 b** as well as their isotopomers were prepared according to literature procedures.^21^ Matrix isolation experiments were performed by standard techniques using two‐staged closed‐cycle helium cryostats (cooling power 1 W at 4 K) to obtain temperatures around 3 K. The matrices were generated by co‐deposition of (*d_1_‐/CD_3_‐)* **2 a/b** with a large excess of argon on top of a cold CsI window at 3 K.

### Computational details

Gas‐phase geometry optimizations and frequency calculations were performed using the B3LYP functional^36^ employing the 6–311G(d,p) basis set^37^ as implemented in Gaussian 09.^38^ The SCT calculations,^39^ performed via the Gaussrate^40^ and Polyrate^41^ software packages, utilized the D3 dispersion correction^42^ in combination with the computationally less demanding 6‐31G(d) basis set in order to allow for efficient calculations of the semibullvalenes’ potential energy surfaces. For an extended discussion of the methodology, see Supporting Information.

## Conflict of interest

The authors declare no conflict of interest.

## Supporting information

As a service to our authors and readers, this journal provides supporting information supplied by the authors. Such materials are peer reviewed and may be re‐organized for online delivery, but are not copy‐edited or typeset. Technical support issues arising from supporting information (other than missing files) should be addressed to the authors.

Supplementary

## References

[1] D. G. Truhlar , J. Phys. Org. Chem. 2010, 23, 660–676.

[2a] H. Inui , K. Sawada , S. Oishi , K. Ushida , R. J. McMahon , J. Am. Chem. Soc. 2013, 135, 10246–10249; 23795602 10.1021/ja404172s

[2b] C. M. Nunes , I. Reva , S. Kozuch , R. J. McMahon , R. Fausto , J. Am. Chem. Soc. 2017, 139, 17649–17659; 29112415 10.1021/jacs.7b10495

[2c] C. M. Nunes , A. K. Eckhardt , I. Reva , R. Fausto , P. R. Schreiner , J. Am. Chem. Soc. 2019, 141, 14340–14348; 31423776 10.1021/jacs.9b06869

[2d] T. Schleif , J. Mieres-Perez , S. Henkel , E. Mendez-Vega , H. Inui , R. J. McMahon , W. Sander , J. Org. Chem. 2019, 84, 16013–16018. 31730349 10.1021/acs.joc.9b02482

[3a] A. Datta , D. A. Hrovat , W. T. Borden , J. Am. Chem. Soc. 2008, 130, 6684–6685; 18447359 10.1021/ja801089p

[3b] O. M. Gonzalez-James , X. Zhang , A. Datta , D. A. Hrovat , W. T. Borden , D. A. Singleton , J. Am. Chem. Soc. 2010, 132, 12548–12549. 20722415 10.1021/ja1055593 PMC2935944

[4] T. Schleif , J. Mieres-Perez , S. Henkel , M. Ertelt , W. T. Borden , W. Sander , Angew. Chem. Int. Ed. 2017, 56, 10746–10749; 10.1002/anie.201704787 28643896

[5] X. Zhang , D. A. Hrovat , W. T. Borden , Org. Lett. 2010, 12, 2798–2801. 20507087 10.1021/ol100879t

[6] B. K. Carpenter , J. Am. Chem. Soc. 1983, 105, 1700–1701.

[7] J. H. Baraban , M.-A. Martin-Drumel , P. B. Changala , S. Eibenberger , M. Nava , D. Patterson , J. F. Stanton , G. B. Ellison , M. C. McCarthy , Angew. Chem. Int. Ed. 2018, 57, 1821–1825; 10.1002/anie.201709966 29239124

[8a] S. L. Buchwalter , G. L. Closs , J. Am. Chem. Soc. 1975, 97, 3857–3858;

[8b] S. L. Buchwalter , G. L. Closs , J. Am. Chem. Soc. 1979, 101, 4688–4694.

[9] M. B. Sponsler , R. Jain , F. D. Coms , D. A. Dougherty , J. Am. Chem. Soc. 1989, 111, 2240–2252.

[10] Z. Wu , R. Feng , H. Li , J. Xu , G. Deng , M. Abe , D. Bégué , K. Liu , X. Zeng , Angew. Chem. Int. Ed. 2017, 56, 15672–15676; 10.1002/anie.201710307 29063647

[11] P. Zuev , R. S. Sheridan , J. Am. Chem. Soc. 1994, 116, 4123–4124.

[12] P. S. Zuev , R. S. Sheridan , T. V. Albu , D. G. Truhlar , D. A. Hrovat , W. T. Borden , Science 2003, 299, 867–870. 12574623 10.1126/science.1079294

[13] R. A. Moss , R. R. Sauers , R. S. Sheridan , J. Tian , P. S. Zuev , J. Am. Chem. Soc. 2004, 126, 10196–10197. 15315403 10.1021/ja0488939

[14a] W. Sander , G. Bucher , F. Reichel , D. Cremer , J. Am. Chem. Soc. 1991, 113, 5311–5322;

[14b] M. Ertelt , D. A. Hrovat , W. T. Borden , W. Sander , Chem. Eur. J. 2014, 20, 4713–4720. 24616081 10.1002/chem.201303792

[15] D. Moskau , R. Aydin , W. Leber , H. Günther , H. Quast , H. D. Martin , K. Hassenrück , L. S. Miller , K. Grohmann , Chem. Ber. 1989, 122, 925–931.

[16] C. Schnieders , K. Müllen , C. Braig , H. Schuster , J. Sauer , Tetrahedron Lett. 1984, 25, 749–752.

[17] A. K. Cheng , F. A. L. Anet , J. Mioduski , J. Meinwald , J. Am. Chem. Soc. 1974, 96, 2887–2891.

[18] I. Sellner , H. Schuster , H. Sichert , J. Sauer , H. Nöth , Chem. Ber. 1983, 116, 3751–3761.

[19] J. Stapersma , P. Kuipers , G. W. Klumpp , Rec. Trav. Chim. 2010, 101, 213–218.

[20] R. Askani , H.-O. Kalinowski , B. Weuste , Org. Magn. Res. 1982, 18, 176–177.

[21] H. Quast , Y. Görlach , E.-M. Peters , K. Peters , H. G. von Schnering , L. M. Jackman , G. Ibar , A. J. Freyer , Chem. Ber. 1986, 119, 1801–1835.

[22] Y. C. Wang , S. H. Bauer , J. Am. Chem. Soc. 1972, 94, 5651–5657.

[23] W. Siebrand , T. A. Wildman , Acc. Chem. Res. 1986, 19, 238–243.

[24] W. T. Borden , Wiley Interdiscip. Rev.: Comput. Mol. Sci. 2016, 6, 20–46.

[25] S. Kozuch , Phys. Chem. Chem. Phys. 2014, 16, 7718–7727. 24590008 10.1039/c4cp00115j

[26] L. S. Miller , K. Grohmann , J. J. Dannenberg , J. Am. Chem. Soc. 1983, 105, 6862–6865.

[27a] Z. Bacic , D. Benoit , M. Biczysko , J. Bowman , S. Bradforth , T. Burd , G. Chambaud , D. Clary , C. Crépin , M. Dracinsky , P. Felker , I. Fischer , F. Gianturco , M. Hochlaf , K. Kouril , I. Kratochvilova , C. Liu , A. McCoy , J. Miyazaki , H. Mouhib , J. Richardson , P. Slaviček , T. Stoecklin , K. Szalewicz , A. van der Avoird , A. Zehnacker-Rentien , Faraday Discuss. 2018, 212, 569–601; 30520925 10.1039/c8fd90053a

[27b] A. Gutiérrez-Quintanilla , M. Chevalier , J. Ceponkus , R. R. Lozada-García , J.-M. Mestdagh , C. Crépin , Faraday Discuss. 2018, 212, 499–515; 30229772 10.1039/c8fd00080h

[27c] A. Gutiérrez-Quintanilla , M. Chevalier , R. Platakyte , J. Ceponkus , C. Crépin , Phys. Chem. Chem. Phys. 2020, 22, 6115–6121. 32096505 10.1039/c9cp06866j

[28] M. Seefelder , M. Heubes , H. Quast , W. D. Edwards , J. R. Armantrout , R. V. Williams , C. J. Cramer , A. C. Goren , D. A. Hrovat , W. T. Borden , J. Org. Chem. 2005, 70, 3437–3449. 15844976 10.1021/jo0502089

[29a] A. Benesi , R. Bertermann , H. Förster , M. Heubes , L. M. Jackman , T. Koritsanszky , P. Luger , A. Mayer , H. Quast , M. Seefelder , D. Zobel , J. Am. Chem. Soc. 2000, 122, 4455–4463;

[29b] H. Quast , J. Carlsen , R. Janiak , E. M. Peters , K. Peters , H. G. Von Schnering , Chem. Ber. 1992, 125, 955–968;

[29c] L. M. Jackman , A. Benesi , A. Mayer , H. Quast , E. M. Peters , K. Peters , H. G. Von Schnering , J. Am. Chem. Soc. 1989, 111, 1512–1513.

[30] P. R. Griffiths , D. E. Pivonka , R. V. Williams , Chem. Eur. J. 2011, 17, 9193–9199. 21735493 10.1002/chem.201100025

[31a] N. Akai , S. Kudoh , M. Nakata , J. Phys. Chem. A 2003, 107, 3655–3659;

[31b] N. Akai , S. Kudoh , M. Takayanagi , M. Nakata , Chem. Phys. Lett. 2002, 356, 133–139;

[31c] N. Akai , S. Kudoh , M. Nakata , J. Phys. Chem. A 2003, 107, 2635–2641;

[31d] N. Akai , S. Kudoh , M. Takayanagi , M. Nakata , J. Phys. Chem. A 2002, 106, 11029–11033.

[32] D. W. Firth , P. F. Barbara , H. P. Trommsdorff , Chem. Phys. 1989, 136, 349–360.

[33] R. L. Redington , J. Chem. Phys. 1990, 92, 6447–6455.

[34] T. Bredtmann , J. Manz , J.-M. Zhao , J. Phys. Chem. A 2016, 120, 3142–3154. 26799383 10.1021/acs.jpca.5b11295

[35] R. Askani , H.-O. Kalinowski , B. Pelech , B. Weuste , Tetrahedron Lett. 1984, 25, 2321–2324.

[36a] A. D. Becke , J. Chem. Phys. 1993, 98, 5648–5652;

[36b] C. Lee , W. Yang , R. G. Parr , Phys. Rev. B 1988, 37, 785–789. 10.1103/physrevb.37.785 9944570

[37] R. Krishnan , J. S. Binkley , R. Seeger , J. A. Pople , J. Chem. Phys. 1980, 72, 650–654.

[38] Gaussian 09, Revision A.01, M. J. Frisch, G. W. Trucks, H. B. Schlegel, G. E. Scuseria, M. A. Robb, J. R. Cheeseman, G. Scalmani, V. Barone, B. Mennucci, G. A. Petersson, H. Nakatsuji, M. Caricato, X. Li, H. P. Hratchian, A. F. Izmaylov, J. Bloino, G. Zheng, J. L. Sonnenberg, M. Hada, M. Ehara, K. Toyota, R. Fukuda, J. Hasegawa, M. Ishida, T. Nakajima, Y. Honda, O. Kitao, H. Nakai, T. Vreven, J. A. Montgomery, Jr., J. E. Peralta, F. Ogliaro, M. J. Bearpark, J. Heyd, E. N. Brothers, K. N. Kudin, V. N. Staroverov, R. Kobayashi, J. Normand, K. Raghavachari, A. P. Rendell, J. C. Burant, S. S. Iyengar, J. Tomasi, M. Cossi, N. Rega, N. J. Millam, M. Klene, J. E. Knox, J. B. Cross, V. Bakken, C. Adamo, J. Jaramillo, R. Gomperts, R. E. Stratmann, O. Yazyev, A. J. Austin, R. Cammi, C. Pomelli, J. W. Ochterski, R. L. Martin, K. Morokuma, V. G. Zakrzewski, G. A. Voth, P. Salvador, J. J. Dannenberg, S. Dapprich, A. D. Daniels, Ö. Farkas, J. B. Foresman, J. V. Ortiz, J. Cioslowski, D. J. Fox, Gaussian, Inc., Wallingford, CT, USA, **2009**.

[39a] Y. P. Liu , G. C. Lynch , T. N. Truong , D. H. Lu , D. G. Truhlar , B. C. Garrett , J. Am. Chem. Soc. 1993, 115, 2408–2415;

[39b] D.-h. Lu , T. N. Truong , V. S. Melissas , G. C. Lynch , Y.-P. Liu , B. C. Garrett , R. Steckler , A. D. Isaacson , S. N. Rai , G. C. Hancock , J. G. Lauderdale , T. Joseph , D. G. Truhlar , Comput. Phys. Commun. 1992, 71, 235–262.

[40] J. Zheng, J. L. Bao, S. Zhang, J. C. Corchado, R. Meana-Pañeda, Y.-Y. Chuang, E. L. Coitiño, B. A. Ellingson, D. G. Truhlar in *GAUSSRATE 17*, *Vol* University of Minnesota, Minneapolis, **2017**.

[41] J. Zheng, J. L. Bao, R. Meana-Pañeda, S. Zhang, B. J. Lynch, J. C. Corchado, Y.-Y. Chuang, P. L. Fast, W.-P. Hu, Y.-P. Liu, G. C. Lynch, K. A. Nguyen, C. F. Jackels, A. F. Ramos, B. A. Ellingson, V. S. Melissas, J. Villà, I. Rossi, E. L. Coitiño, T. V. A. J. Pu, A. Ratkiewicz, R. Steckler, B. C. Garrett, A. D. Isaacson, D. G. Truhlar in *POLYRATE Version 2016–2A*, *Vol* University of Minnesota, Minneapolis, **2016**.

[42] S. Grimme , J. Antony , S. Ehrlich , H. Krieg , J. Chem. Phys. 2010, 132, 154104. 20423165 10.1063/1.3382344

