# Supplementary material for: Heavy‐Atom Tunneling in Semibullvalenes: How Driving Force, Substituents, and Environment Influence the Tunneling Rates
Source: Chemistry. 2020 Jul 28;26(46):10452–8. doi: 10.1002/chem.202001202 (PMC7496793; doi:10.1002/chem.202001202)
Supplement: Supplementary file 1 — Supplementary [file CHEM-26-10452-s001.pdf]

Chemistry–A European Journal

Supporting Information

Heavy-Atom Tunneling in Semibullvalenes: How Driving Force, Substituents, and Environment Influence the Tunneling Rates

Tim Schleif,[a] Jörg Tatchen,[b] Julien F. Rowen,[a] Frederike Beyer,[a] Elsa Sanchez-Garcia,*[b] and Wolfram Sander*[a]

Supporting Information

Contents

IR spectroscopic data 2

Experimental kinetic data 4

Calculated kinetic data 5

Synthesis 10

Isotopomeric ratios 12

Z-Matrices 13

Literature references 21

IR spectroscopic data

Table S1. IR spectroscopic data of *CD_3^{cyc}*-1,5-dimethylsemibullvalene (*CD_3^{cyc}*-**2a**).

Mode	Calculated[a] $\tilde{\nu}$/cm^{-1} (I_{abs})	Neon[b] $\tilde{\nu}$/cm^{-1} (I_{rel})	Assignment[c]
20	829.7 (10)	816.1 (16)	C-H twist.
21	859.0 (18)	842.9 (100)	C-H wag.
41	1373.0 (16)	1349.4 (9)	C-H rock.
46	1497.9 (7)	1457.2 (12)	C-H scis.

a: Calculated at B3LYP/6-311G(d,p) level of theory. b: Neon matrix at 3 K. c: Tentative assignment.

Table S2. IR spectroscopic data of *CD_3^{open}*-1,5-dimethylsemibullvalene (*CD_3^{open}*-**2a**).

Mode	Calculated[a] $\tilde{\nu}$/cm^{-1} (I_{abs})	Neon[b] $\tilde{\nu}$/cm^{-1} (I_{rel})	Assignment[c]
20	818.4 (15)	802.1 (43)	C-H rock.
21	860.6 (15)	847.6 (100)	C-H wag.
24	946.7 (7)	916.5 (15)	C-H twist.
25	959.5 (8)	944.1 (8)	C-H wag.
39	1248.4 (7)	1242.0 (9)	C-H wag.
41	1374.8 (13)	1352.4 (16)	C-H rock.
45	1489.8 (6)	1447.9 (10)	C-H twist.
46	1499.8 (10)	1464.7 (16)	C-H scis.

a: Calculated at B3LYP/6-311G(d,p) level of theory. b: Neon matrix at 3 K. c: Tentative assignment

Table S3. IR spectroscopic data of 3,7-dicyano-1,5-dimethylsemibullvalene (**2b**).

Mode	Calculated[a] $\tilde{\nu}$/cm^{-1} (I_{abs})	Argon[b] $\tilde{\nu}$/cm^{-1} (I_{rel})	Assignment[c]
16	541.0 (3)	524.1 (10)	C-C rock.
19	570.1 (7)	548.7 (12)	C-C rock.
20	596.5 (7)	574.7 (21)	C-C stretch.
25	863.9 (7)	838.5 (20)	C-H rock.
26	871.2 (34)	849.6 (82)	C-H wag.
27	881.3 (17)	852.9 (31)	C-H scis.
29	922.9 (5)	911.9 (11)	C-H rock.
30	933.2 (6)	922.8 (11)	C-H wag.
32	981.1 (6)	959.6 (13)	C-H wag.
44	1319.6 (13)	1302.8 (43)	C-H scis.
50	1495.3 (18)	1454.3 (41)	C-H (CH_3) wag.
51	1502.1 (15)	1457.1 (37)	C-H (CH_3) twist.
52	1507.4 (2)	1465.6 (11)	C-H (CH_3) wag.
54	1622.3 (6)	1599.3 (2)	C=C stretch.
55	2334.1 (19)	2233.6 (100)	C≡N str.

a: Calculated at B3LYP/6-311G(d,p) level of theory. b: Argon matrix at 9 K. c: Tentative assignment.

Table S4. IR spectroscopic data of *d*^{4}-3,7-dicyano-1,5-dimethylsemibullvalene (*d*^{4}-**2b**).

Mode	Calculated[a] $\tilde{\nu}$/cm^{-1} (I_{abs})	Neon[b] $\tilde{\nu}$/cm^{-1} (I_{rel})	Argon[b] $\tilde{\nu}$/cm^{-1} (I_{rel})	Nitrogen[b] $\tilde{\nu}$/cm^{-1} (I_{rel})	Xenon[b] $\tilde{\nu}$/cm^{-1} (I_{rel})	Assignment[c]
21	637.5 (9)	624.1 (29)	621.9 (96)	625.3 (33)	621.3 (100)	C-H wag.
23	680.9 (14)	663.3 (100)		663.0 (100)		C-H rock.
25	764.3 (4)	739.5 (20)	737.9 (79)	741.8 (10)	736.1 (57)	C-C wag.
26	828.9 (7)	816.8 (56)	816.7 (100)	818.2 (86)	815.8 (72)	C-H wag.
32	979.8 (8)	960.3 (10)				C-H wag.
44	1300.7 (11)	1286.4 (90)	1285.1 (82)	1286.4 (33)	1285.0 (45)	C-H scis.

a: Calculated at B3LYP/6-311G(d,p) level of theory. b: Matrices at 3 K. c: Tentative assignment.

Table S5. IR spectroscopic data of *d*^{2}-3,7-dicyano-1,5-dimethylsemibullvalene (*d*^{2}-**2b**).

Mode	Calculated[a] $\tilde{\nu}$/cm^{-1} (I_{abs})	Neon[b] $\tilde{\nu}$/cm^{-1} (I_{rel})	Argon[b] $\tilde{\nu}$/cm^{-1} (I_{rel})	Nitrogen[c] $\tilde{\nu}$/cm^{-1} (I_{rel})	Xenon[b] $\tilde{\nu}$/cm^{-1} (I_{rel})	Assignment[c]
21	644.7 (6)	628.0 (72)		629.3 (29)		C-H wag.
23	698.4 (8)	680.8 (35)	680.1 (58)	680.5 (29)		C-H rock.
25	773.8 (10)	754.6 (45)	752.2 (60)	754.2 (40)	751.1 (100)	C-H rock.
28	879.0 (37)	851.1 (100)	849.9 (76)	855.0 (100)	848.1 (84)	C-H wag.
44	1316.8 (12)	1300.8 (45)	1299.7 (100)		1296.4 (70)	C-H scis.

a: Calculated at B3LYP/6-311G(d,p) level of theory. b: Matrices at 3 K. c: Tentative assignment.

IR spectroscopic data for *d*^{4}-**2b** + *d*^{2}-**2b** (+ **2b**):

IR (neat, 3 K): $\tilde{\nu}$ = 2227 vs, 1571 m, 1454 s, 1300 m, 1285 m, 1261 w, 1193 w, 1098 m, 865 s, 650 sh, 816 m, 751 m, 742 sh, 661 s, 627 m, 572 s cm-1.

IR (*p*-H_2 matrix, 3 K): $\tilde{\nu}$ (I_{rel}) = 2227.9 (100), 1571.7 (14), 1454.0 (55), 1299.8 (11), 1285.8 (6), 1261.1 (4), 1191.8 (4), 1099.2 (6), 864.5 (54), 850.0 (14), 816.2 (23), 752.0 (8), 740.6 (4), 662.3 (24), 627.5 (12), 571.9 (14) cm^{-1}.

Table S6. IR spectroscopic data of *CD$_3$^{cyc}*-3,7-dicyano-1,5-dimethylsemibullvalene (*CD$_3$^{cyc}*-**2b**).

Mode	Calculated[a] $\tilde{\nu}$/cm^{-1} (I_{abs})	Neon[b] $\tilde{\nu}$/cm^{-1} (I_{rel})	Assignment[c]
28	869.9 (30)	850.7 (13)	C-H wag.
29	881.9 (23)	857.0 (42)	C-H wag.
30	922.2 (6)	912.3 (35)	C-H rock.
31	933.1 (5)	922.5 (28)	C-H wag.
32	960.3 (2)	946.1 (32)	C-H wag.
47	1316.0 (14)	1298.6 (32)	C-H scis.
49	1415.2 (5)	1384.0 (76)	C-H scis.
50	1438.6 (8)	1418.6 (38)	C-H (CH_3) wag.
52	1500.1 (8)	1459.2 (100)	C-H (CH_3) scis.

a: Calculated at B3LYP/6-311G(d,p) level of theory. b: Neon matrix at 3 K. c: Tentative assignment

Table S7. IR spectroscopic data of CD_3^{open}-3,7-dicyano-1,5-dimethylsemibullvalene (CD_3^{open}-**2b**).

Mode	Calculated[a] $\tilde{\nu}$/cm^{-1} (I_{abs})	Neon[b] $\tilde{\nu}$/cm^{-1} (I_{rel})	Assignment
28	871.9 (24)	853.7 (19)	C-H wag.
29	883.7 (28)	866.0 (75)	C-H wag.
30	892.6 (5)	881.5 (72)	C-H rock.
32	974.5 (9)	954.5 (100)	C-H wag.
34	1027.5 (2)	1014.4 (8)	C-H (CH_3) wag.
36	1069.4 (4)	1047.6 (7)	C-H (CH_3) scis.
37	1074.7 (4)	1053.3 (27)	C-H (CH_3) scis.
43	1194.3 (4)	1185.2 (22)	C-H scis.
47	1319.5 (14)	1307.9 (34)	C-H scis.
50	1440.0 (10)	1428.6 (36)	C-H (CH_3) wag.
51	1493.0 (7)	1454.4 (14)	C-H (CH_3) scis.

a: Calculated at B3LYP/6-311G(d,p) level of theory. b: Neon matrix at 3 K. c: Tentative assignment

Experimental kinetic data

Table S8. Rate constants and apparent half-lives as fitted to equation 1 with β as variable for the Cope rearrangement of d_1-1,5-dimethyl-3,7-dicyanosemibullvalene (d_1-**2b**) via QMT.

Matrix	T / K	k / 10^{-5} s^{-1}	τ_{app} / h	t_{final} / h	β
N_2	3	5.7 ± 1.1	3.9	41.0	0.7 ± 0.1
N_2	8	4.7 ± 0.9	3.5	30.0	0.7 ± 0.0
N_2	13	15.3 ± 6.7	0.7	38.4	0.4 ± 0.1
N_2	18	6.3 ± 1.0	2.6	41.3	0.7 ± 0.1
N_2	23	*fit does not converge with β as variable*			
Ne	3	1.9 ± 0.7	10.1	52.7	1.0 ± 0.4
Ne	3	3.0 ± 0.3	6.4	36.0	1.0 ± 0.1
Ne	6	4.3 ± 0.8	4.5	39.5	1.0 ± 0.3
Ne	6	2.0 ± 1.0	9.6	30.7	1.0 ± 0.5
Ar	3	5.8 ± 2.1	2.3	39.9	0.5 ± 0.1
Ar	25	7.5 ± 2.0	2.6	26.6	1.0 ± 0.4
Xe	3	2.1 ± 1.9	2.3	39.2	0.5 ± 0.2
Xe	35	3.8 ± 0.8	2.3	38.5	0.8 ± 0.2

Table S9. Rate constants and apparent half-lives as fitted to equation 1 with β as variable for the Cope rearrangement of CD_3-1,5-dimethylsemibullvalene (CD_3-**2a**) in a neon matrix via QMT.

T / K	k / 10^{-4} s^{-1}	τ_{app} / h	t_{final} / h	β
3	2.3 ± 0.6	0.7	16.4	0.72 ± 0.07
3	2.0 ± 0.5	0.7	14.1	0.65 ± 0.14
6	1.0 ± 0.2	1.5	17.0	0.63 ± 0.12
6	1.3 ± 0.9<br>(0.09 ± 0.27)[a]	0.8<br>(7.9)	17.6<br>(68.5)	0.39 ± 0.12<br>(0.26 ± 0.08)[a]

a: For better comparability, the kinetic data was also evaluated for a shorter duration of the measurement.

Table S10. Rate constants and apparent half-lives as fitted to equation 1 with β as variable for the Cope rearrangement of *CD₃*-1,5-dimethyl-3,7-dicyanosemibullvalene (*CD₃*-**2b**) in a neon matrix via QMT.

T / K	k / 10^{-5} s^{-1}	τ_{app} /h	t_{final} / h	*β*
3	5.0 ± 0.6<br>(9.1 ± 0.8)	1.8<br>(1.3)	50.8<br>(94.2)	0.33 ± 0.02<br>(0.44 ± 0.03)[a]
3	11.7 ± 1.7	0.9	47.5	0.40 ± 0.04
6	7.9 ± 0.9	1.4	50.8	0.39 ± 0.03
6	6.8 ± 0.9	1.4	63.8	0.34 ± 0.03

a: For better comparability, the kinetic data was also evaluated for a shorter duration of the measurement.

Calculated kinetic data

Methodology

In order to compute low-temperature tunneling rates for the SBV derivatives, we employed transition state theory (TST) together with the small-curvature tunneling (SCT) approximation[1] as implemented in the Polyrate[2] and Gaussrate[3] program packages.

The Gaussian09 Rev. D01[4] suite of programs was employed in the quantum-chemical parts of the calculations, i.e., for the optimization of stationary points (minima of reactants and products as well as the transition states) and for the on-the-fly determination of the reaction path (minimum energy path, MEP) in Gaussrate. We used closed-shell Kohn-Sham density functional theory (DFT) with the B3LYP functional[5] and 6-31G(d)[6] basis sets, following Ref.[7] In addition, GD3 dispersion corrections[8] were taken into account.

Because tunneling rates are known be rather sensitive to minor changes of the underlying potential energy surface (PES), we devised the following measures in order to increase the accuracy of the calculated potential energy profile:

1. Throughout, DFT calculations with the Gaussian09 program are performed with a convergence criterion of 10-9 for the density matrix and with integration grids chosen as 'UltraFine'.

2. Geometries of stationary points are calculated in advance imposing maximal point group symmetry (Cs for reactants and products and C2v for transition states, TS). Moreover, we requested 'VeryTight' convergence for the geometry optimizations. The optimized geometries are supplied to the Gaussrate/Polyrate programs as input. Their status is assigned as optimized ('STATUS 2'). I.e., within the Gaussrate runs these geometries are not reoptimized via Gaussian on-the-fly calculations. This way, the stationary points exhibit the symmetry of the tunneling problem except for rather tiny numerical differences between the educt and product optimization results.

3. Within the Gaussrate runs, all Gaussian09 calculations which are performed on-the-fly during the integration of the reaction path are carried out without point group symmetry ('Symmetry=None') in order to avoid reorientation of the molecule or energetic artifacts due to flips of an automatically determined point group symmetry.

(We note that direct optimization of the stationary points by Gaussrate is also possible. When the request for disabling symmetry also applied for the geometry optimization, a minor, but noticeable asymmetry of the PES profile along the reaction path resulted for many cases, however. The latter caused an undesirable asymmetry between the calculated forward and backward tunneling rates, in particular for the low temperature regime. The first approach as described above yields approximately equal rates for forward and backward tunneling in all cases and thus was considered preferable.)

The Page-McIver algorithm[9] of Polyrate is employed in order to integrate the MEP underlying the calculation of tunneling coefficients within the SCT calculation. The step size was chosen as 'SSTEP' = 10-2 a0. In every step of the MEP, both gradients and Hessians were recalculated by direct calculation (no interpolation).

The MEP was followed from the TS (corresponding to a value s=0 of the reaction path length variable) towards the valleys of both reactant (s<0) and product (s>0). The MEP was calculated for an interval [-2.0 Å; 2.0 Å] for all compounds except for the parent semibullvalene **22**. For the latter, only the interval [-1.8 Å; 1.8 Å] was accessible whereas attempts with larger intervals did not converge. We verified by means of the resulting energy range of the MEPs and by inspecting molecular geometries along the paths that these intervals are sufficiently large to effectively cover the entire MEPs from the TS to the reactant and product minima.

Table S11. Rate constants for the Cope rearrangement of semibullvalene (**22**) calculated via the SCT methodology at the B3LYP-D3/6-31G(d) level of theory.

T / K	forward		backward		averaged	
	TST+SCT / s^{-1}	CVT+SCT / s^{-1}	TST+SCT / s^{-1}	CVT+SCT / s^{-1}	TST+SCT / s^{-1}	CVT+SCT / s^{-1}
10	1.36E-04	1.36E-04	1.35E-04	1.35E-04	1.36E-04	1.36E-04
20	3.21E-04	3.21E-04	3.20E-04	3.20E-04	3.20E-04	3.20E-04
30	6.02E-04	6.02E-04	6.01E-04	6.01E-04	6.02E-04	6.02E-04
40	1.15E-03	1.15E-03	1.15E-03	1.15E-03	1.15E-03	1.15E-03
50	2.72E-03	2.72E-03	2.71E-03	2.71E-03	2.71E-03	2.71E-03
60	1.44E-02	1.44E-02	1.44E-02	1.44E-02	1.44E-02	1.44E-02
70	3.85E-01	3.85E-01	3.84E-01	3.84E-01	3.84E-01	3.84E-01
80	1.13E+01	1.13E+01	1.13E+01	1.13E+01	1.13E+01	1.13E+01
90	1.90E+02	1.90E+02	1.90E+02	1.90E+02	1.90E+02	1.90E+02
100	1.95E+03	1.95E+03	1.95E+03	1.95E+03	1.95E+03	1.95E+03
125	1.43E+05	1.43E+05	1.43E+05	1.43E+05	1.43E+05	1.43E+05
150	2.69E+06	2.69E+06	2.69E+06	2.69E+06	2.69E+06	2.69E+06
175	2.28E+07	2.28E+07	2.28E+07	2.28E+07	2.28E+07	2.28E+07
200	1.16E+08	1.16E+08	1.16E+08	1.16E+08	1.16E+08	1.16E+08
225	4.18E+08	4.18E+08	4.18E+08	4.18E+08	4.18E+08	4.18E+08
250	1.18E+09	1.18E+09	1.18E+09	1.18E+09	1.18E+09	1.18E+09
275	2.78E+09	2.78E+09	2.78E+09	2.78E+09	2.78E+09	2.78E+09
300	5.72E+09	5.72E+09	5.72E+09	5.72E+09	5.72E+09	5.72E+09

Table S12. Rate constants for the Cope rearrangement of 1,5-dimethylsemibullvalene (**2a**) calculated via the SCT methodology at the B3LYP-D3/6-31G(d) level of theory.

T / K	forward		backward		averaged	
	TST+SCT / s^{-1}	CVT+SCT / s^{-1}	TST+SCT / s^{-1}	CVT+SCT / s^{-1}	TST+SCT / s^{-1}	CVT+SCT / s^{-1}
10	1.72E-03	1.72E-03	1.83E-03	1.83E-03	1.78E-03	1.78E-03
20	4.79E-03	4.79E-03	4.94E-03	4.94E-03	4.86E-03	4.86E-03
30	1.01E-02	1.01E-02	1.03E-02	1.03E-02	1.02E-02	1.02E-02
40	2.09E-02	2.09E-02	2.12E-02	2.12E-02	2.11E-02	2.11E-02
50	5.93E-02	5.93E-02	6.00E-02	6.00E-02	5.96E-02	5.96E-02
60	6.57E-01	6.57E-01	6.64E-01	6.64E-01	6.60E-01	6.60E-01
70	2.15E+01	2.15E+01	2.17E+01	2.17E+01	2.16E+01	2.16E+01
80	4.47E+02	4.47E+02	4.51E+02	4.51E+02	4.49E+02	4.49E+02
90	5.23E+03	5.23E+03	5.27E+03	5.27E+03	5.25E+03	5.25E+03
100	3.91E+04	3.91E+04	3.93E+04	3.93E+04	3.92E+04	3.92E+04
125	1.58E+06	1.58E+06	1.59E+06	1.59E+06	1.58E+06	1.58E+06
150	1.96E+07	1.96E+07	1.97E+07	1.97E+07	1.97E+07	1.97E+07
175	1.23E+08	1.23E+08	1.23E+08	1.23E+08	1.23E+08	1.23E+08
200	4.94E+08	4.94E+08	4.97E+08	4.97E+08	4.95E+08	4.95E+08
225	1.48E+09	1.48E+09	1.49E+09	1.49E+09	1.49E+09	1.49E+09
250	3.61E+09	3.61E+09	3.63E+09	3.63E+09	3.62E+09	3.62E+09
275	7.53E+09	7.53E+09	7.57E+09	7.57E+09	7.55E+09	7.55E+09
300	1.40E+10	1.40E+10	1.41E+10	1.41E+10	1.40E+10	1.40E+10

Table S13. Rate constants calculated for the Cope rearrangement of 3,7-dicyano-1,5-dimethylsemibullvalene (**2b**) via the SCT methodology at the B3LYP-D3/6-31G(d) level of theory.

T / K	forward		backward		averaged	
	TST+SCT / s^{-1}	CVT+SCT / s^{-1}	TST+SCT / s^{-1}	CVT+SCT / s^{-1}	TST+SCT / s^{-1}	CVT+SCT / s^{-1}
10	1.54E-04	1.54E-04	1.46E-04	1.46E-04	1.50E-04	1.50E-04
20	3.73E-04	3.73E-04	3.65E-04	3.65E-04	3.69E-04	3.69E-04
30	7.12E-04	7.12E-04	7.01E-04	7.01E-04	7.06E-04	7.06E-04
40	1.28E-03	1.28E-03	1.27E-03	1.27E-03	1.27E-03	1.27E-03
50	2.40E-03	2.40E-03	2.37E-03	2.37E-03	2.39E-03	2.39E-03
60	5.21E-03	5.21E-03	5.17E-03	5.17E-03	5.19E-03	5.19E-03
70	1.62E-02	1.62E-02	1.61E-02	1.61E-02	1.62E-02	1.62E-02
80	1.02E-01	1.02E-01	1.01E-01	1.01E-01	1.01E-01	1.01E-01
90	1.15E+00	1.15E+00	1.14E+00	1.14E+00	1.14E+00	1.14E+00
100	1.31E+01	1.31E+01	1.30E+01	1.30E+01	1.30E+01	1.30E+01
125	1.82E+03	1.82E+03	1.81E+03	1.81E+03	1.82E+03	1.82E+03
150	6.15E+04	6.15E+04	6.11E+04	6.11E+04	6.13E+04	6.13E+04
175	8.22E+05	8.22E+05	8.17E+05	8.17E+05	8.20E+05	8.20E+05
200	5.99E+06	5.99E+06	5.96E+06	5.96E+06	5.97E+06	5.97E+06
225	2.88E+07	2.88E+07	2.86E+07	2.86E+07	2.87E+07	2.87E+07
250	1.03E+08	1.03E+08	1.02E+08	1.02E+08	1.03E+08	1.03E+08
275	2.95E+08	2.95E+08	2.94E+08	2.94E+08	2.94E+08	2.94E+08
300	7.17E+08	7.17E+08	7.13E+08	7.13E+08	7.15E+08	7.15E+08

Table S14. Rate constants calculated for the Cope rearrangement of 3,7-difluoro-1,5-dimethylsemibullvalene (**2c**) via the SCT methodology at the B3LYP-D3/6-31G(d) level of theory.

T / K	forward		backward		averaged	
	TST+SCT / s^{-1}	CVT+SCT / s^{-1}	TST+SCT / s^{-1}	CVT+SCT / s^{-1}	TST+SCT / s^{-1}	CVT+SCT / s^{-1}
10	1.57E-08	1.57E-08	1.83E-08	1.83E-08	1.70E-08	1.70E-08
20	3.94E-08	3.94E-08	4.25E-08	4.25E-08	4.09E-08	4.09E-08
30	8.00E-08	8.00E-08	8.42E-08	8.42E-08	8.21E-08	8.21E-08
40	1.74E-07	1.74E-07	1.81E-07	1.81E-07	1.78E-07	1.78E-07
50	4.68E-07	4.68E-07	4.83E-07	4.83E-07	4.75E-07	4.75E-07
60	3.85E-06	3.85E-06	3.95E-06	3.95E-06	3.90E-06	3.90E-06
70	4.04E-04	4.04E-04	4.13E-04	4.13E-04	4.08E-04	4.08E-04
80	3.10E-02	3.10E-02	3.16E-02	3.16E-02	3.13E-02	3.13E-02
90	1.03E+00	1.03E+00	1.05E+00	1.05E+00	1.04E+00	1.04E+00
100	1.79E+01	1.79E+01	1.82E+01	1.82E+01	1.81E+01	1.81E+01
125	3.31E+03	3.31E+03	3.35E+03	3.35E+03	3.33E+03	3.33E+03
150	1.13E+05	1.13E+05	1.14E+05	1.14E+05	1.14E+05	1.14E+05
175	1.46E+06	1.46E+06	1.47E+06	1.47E+06	1.46E+06	1.46E+06
200	1.01E+07	1.01E+07	1.02E+07	1.02E+07	1.02E+07	1.02E+07
225	4.63E+07	4.63E+07	4.66E+07	4.66E+07	4.65E+07	4.65E+07
250	1.58E+08	1.58E+08	1.59E+08	1.59E+08	1.59E+08	1.59E+08
275	4.36E+08	4.36E+08	4.38E+08	4.38E+08	4.37E+08	4.37E+08
300	1.02E+09	1.02E+09	1.03E+09	1.03E+09	1.02E+09	1.02E+09

Table S15. Rate constants calculated for the Cope rearrangement of 3,7-diethinyl-1,5-dimethylsemibullvalene (**2d**) via the SCT methodology at the B3LYP-D3/6-31G(d) level of theory.

T / K	forward		backward		averaged	
	TST+SCT / s^{-1}	CVT+SCT / s^{-1}	TST+SCT / s^{-1}	CVT+SCT / s^{-1}	TST+SCT / s^{-1}	CVT+SCT / s^{-1}
10	4.66E-06	4.66E-06	4.28E-06	4.28E-06	4.47E-06	4.47E-06
20	1.14E-05	1.14E-05	1.10E-05	1.10E-05	1.12E-05	1.12E-05
30	2.20E-05	2.20E-05	2.14E-05	2.14E-05	2.17E-05	2.17E-05
40	4.01E-05	4.01E-05	3.92E-05	3.92E-05	3.96E-05	3.96E-05
50	7.61E-05	7.61E-05	7.48E-05	7.48E-05	7.55E-05	7.55E-05
60	1.70E-04	1.70E-04	1.67E-04	1.67E-04	1.68E-04	1.68E-04
70	5.58E-04	5.58E-04	5.51E-04	5.51E-04	5.55E-04	5.55E-04
80	4.22E-03	4.22E-03	4.17E-03	4.17E-03	4.19E-03	4.19E-03
90	6.58E-02	6.58E-02	6.51E-02	6.51E-02	6.55E-02	6.55E-02
100	1.01E+00	1.01E+00	1.00E+00	1.00E+00	1.01E+00	1.01E+00
125	2.41E+02	2.41E+02	2.39E+02	2.39E+02	2.40E+02	2.40E+02
150	1.15E+04	1.15E+04	1.14E+04	1.14E+04	1.15E+04	1.15E+04
175	1.97E+05	1.97E+05	1.95E+05	1.95E+05	1.96E+05	1.96E+05
200	1.72E+06	1.72E+06	1.71E+06	1.71E+06	1.71E+06	1.71E+06
225	9.53E+06	9.53E+06	9.45E+06	9.45E+06	9.49E+06	9.49E+06
250	3.81E+07	3.81E+07	3.78E+07	3.78E+07	3.80E+07	3.80E+07
275	1.20E+08	1.20E+08	1.19E+08	1.19E+08	1.20E+08	1.20E+08
300	3.15E+08	3.15E+08	3.13E+08	3.13E+08	3.14E+08	3.14E+08

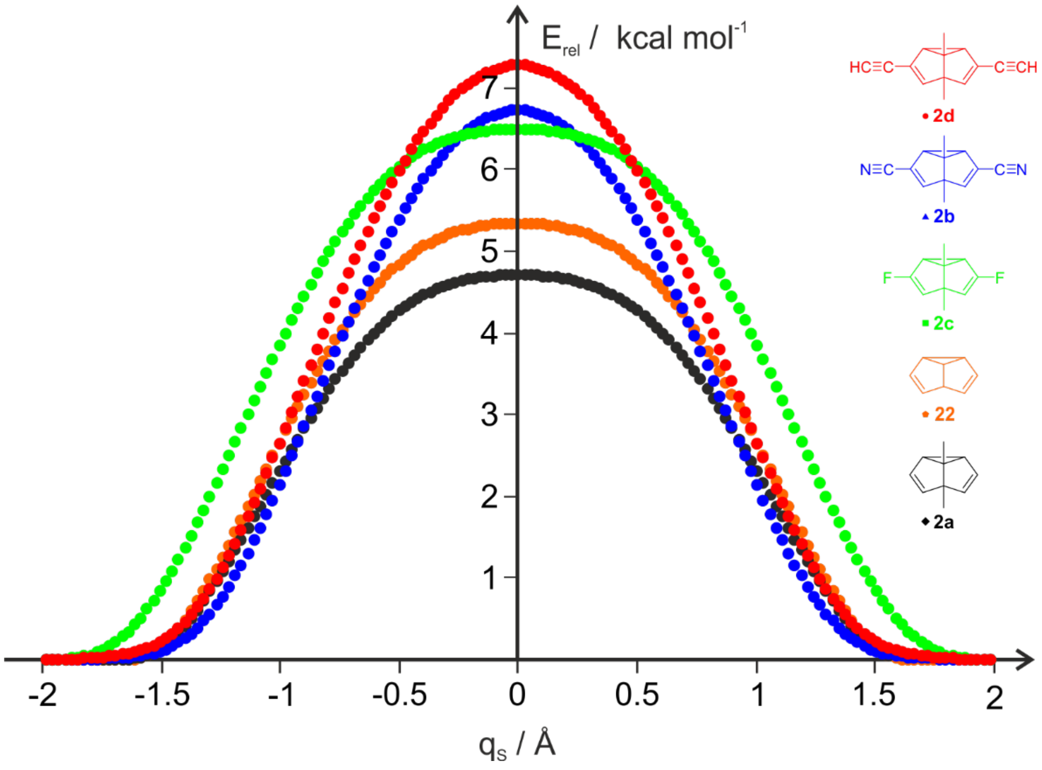

Figure S1. Reaction paths for the investigated semibullvalenes, calculated at the B3LYP-D3/6-31(d) level of theory (without zero-point vibrational correction).

Synthesis

Synthesis of 2,3-butanedione-*d_3* (starting material for the preparation of *CD_3*-semibullvalenes)

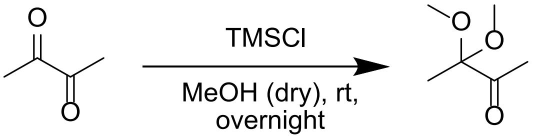

According to a synthesis by Pierau,[10] 100 mL of dry MeOH were added to 7.2 mL (6.2 g, 56.6 mmol) of trimethylsilyl chloride before dropwise addition of 10.2 mL (10.1 g, 117.3 mmol) of 2,3-butanedione at rt. After having stirred at rt overnight, the reaction mixture was poured into a mixture of 100 mL of saturated aqueous $NaHCO_3$ solution and 50 mL of 2 M NaOH solution. The product was extracted with 2 x 100 mL of CH_2Cl_2 and the combined organic extracts were dried over $MgSO_4$. Filtration and removal of the solvents gave 14.0 g (106.0 mmol, 94% yield) of 3,3-dimethoxybutan-2-one as pale yellow liquid.

^{1}H NMR (200 MHz, $CDCl_3$): δ = 3.24 (s, 6H, OCH_3), 2.22 (s, 3H, $COCH_3$), 1.37 (s, 3H, CH_3) ppm.

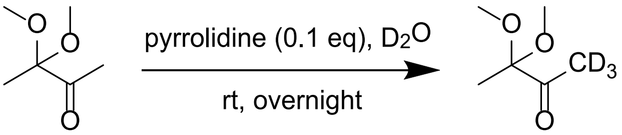

Following a procedure by Chen et al.,[11] 14.0 g (106.0 mmol) of 3,3-dimethoxybutan-2-one were dissolved in 50 mL of D_2O and 0.87 mL (0.75 g, 10.6 mmol) of pyrrolidine were added. After having stirred at rt overnight, the reaction mixture was extracted with 3 x 100 mL of Et_2O. The combined organic extracts were dried over Na_2SO_4 and the solvents were removed to yield 9.0 g (66.6 mmol, 63% yield) of *CD_3*-3,3-dimethoxybutan-2-one as yellow liquid.

^{1}H NMR (200 MHz, $CDCl_3$): δ = 3.21 (s, 6H, OCH_3), 1.34 (s, 3H, CH_3) ppm.

^{13}C NMR (50 MHz, $CDCl_3$): δ = 207.35 (C=O), 102.49 ($C(OMe)_2$), 49.79 (OCH_3), 19.61 ($C(OMe)_2CH_3$) ppm.

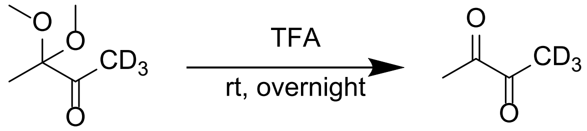

According to instructions by De Kimpe et al.,[12] 8.7 g (64.4 mmol) of *CD_3*-3,3-dimethoxybutan-2-one were dissolved in 16.5 mL (24.4 g, 214.2 mmol) of trifluoroacetic acid and stirred overnight at rt. The reaction mixture was neutralized with saturated aqueous $NaHCO_3$ solution and it was extracted with 4 x 50 mL of CH_2Cl_2. The combined organic extracts were dried over Na_2SO_4, filtered and the solvents were removed at 0 °C by rotary evaporator to obtain 4.8 g (53.9 mmol, 84% yield) of *CD_3*-2,3-butanedione as pale yellow to brown liquid.
^{1}H NMR (200 MHz, $CDCl_3$): δ = 2.33 (s, 3H, CH_3) ppm.

1,5-Dimethylsemibullvalene (**2a**) and 3,7-dicyano-1,5-dimethylsemibullvalene (**2b**) as well as their isotopologues *d^1-/CD_3*-**2a/b** were prepared according to literature procedures;[16] for the generation of *CD_3*-**2a/b** the procedures were slightly modified by the use of *CD_3*-2,3-butanedione as starting material.

<u>1,5-Dimethylsemibullvalene (**2a**)</u>
^{1}H NMR (200 MHz, $CDCl_3$): δ = 5.12 - 5.06 (m, 2H), 4.18 ("dd", 4H, J = 2.5, 1.3 Hz), 1.04 (s, 6H) ppm.
^{13}C NMR (50 MHz, $CDCl_3$): δ = 119.0 (C3/C7), 92.9 (C2/C4/C6/C8), 59.7 (C1/C5), 15.6 (C9/C10) ppm.
<u>*d^1*-1,5-Dimethylsemibullvalene (*d^1*-**2a**)</u>
^{1}H NMR (200 MHz, $CDCl_3$): δ = 5.12 - 5.06 (m, 2H), 4.25 (d, 2H, J = 2.9 Hz), 4.25 (d, 1H, J = 3.7 Hz), 1.04 (s, 6H, 3 CH_3) ppm.
^{13}C NMR (50 MHz, $CDCl_3$): δ = 119.0 (C3/C7), 118.8 (C3/C7), 95.2 (C4/C6), 90.4 (C2/C8), 90.2 (C2/C4, t, J = 25.5 Hz), 59.6 (C1/C5), 59.5 (C1/C5), 15.7 (C9/C10), 15.5 (C9/C10) ppm.
<u>*CD_3*-1,5-Dimethylsemibullvalene (*CD_3*-**2a**)</u>
^{1}H NMR (200 MHz, $CDCl_3$): δ = 5.10 5.12 - 5.06 (m, 2H), 4.29 – 4.06 (m, 4H), 1.04 (s, 3H) ppm.
<u>3,7-Dicyano-1,5-dimethylsemibullvalene (**2b**)</u>
^{1}H NMR (200 MHz, $CDCl_3$): δ = 4.68 (s, 4H), 1.16 (s, 6H) ppm.
<u>*d_1*-3,7-Dicyano-1,5-dimethylsemibullvalene (*d_1*-**2b**)</u>
^{1}H NMR (200 MHz, $CDCl_3$): δ = 4.78 (s, 2H), 4.61 (s, 1H), 1.16 (s, 3H), 1.14 (s, 3H) ppm.
<u>*CD_3*-3,7-dicyano-1,5-dimethylsemibullvalene (*CD_3*-**2b**)</u>
^{1}H NMR (200 MHz, $CDCl_3$): δ = 4.63 (s, 4H), 1.09 (s, 3H) ppm.

<u>Discussion regarding degree of deuteration in *CD_3*-**2a/b**:</u>
The synthetic approach chosen to generate *CD_3*-**2a/b** could also lead to partially deuterated *CD_2H*-**2a/b** as inseparable side products whose exact contribution to the respective sample's composition was hard to determine; similarly, *CDH_2*-**2a/b** could potentially be formed but their presence in the deposited samples was deemed negligible as their generation during the crucial Weiss-Cook condensation (s. below) would require improbable double H/D exchange within the same molecule.

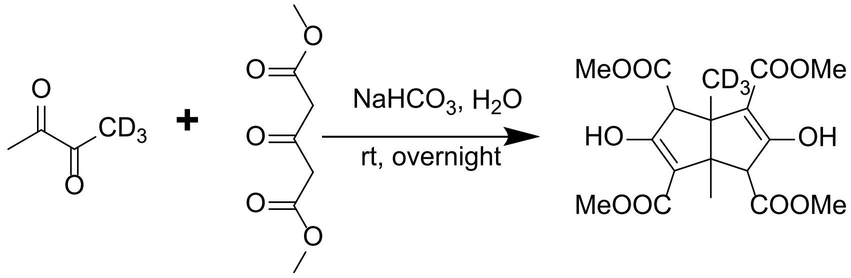

However, the energetic differences between the corresponding isomers of the different isotopologues are all within the same order of magnitude (s. Tab. 4), which allowed for IR spectroscopic investigations without exact analysis or deconvolution of the mixture of different isotopologues present in the samples; for the sake of simplicity, the samples are consistently referred to as *CD_3*-**2a/b** within the paper, as this can reasonably be assumed to represent the majority of the sample.

Isotopomeric ratios

Table S12. Isotopomeric ratio of d^2-**2b** and d^4-**2b** as function of different temperatures and matrix materials. The ratio in neat deposition at 3 K could not be reliably evaluated due to the greater peak width.

T / K	Matrix	d^2-**2b** : d^4-**2b**		predicted ratio[a]	
		t_0	t_{final}	experimental	calculated
3	Ne	1.0	1.1 (52.7 h)	$1.1 \cdot 10^6$	$4.7 \cdot 10^5$
3	Ne	1.0	1.5 (36.0 h)		
6	Ne	1.4	1.6 (39.5 h)	1047	683
6	Ne	1.3	1.6 (30.7 h)		
3	N_2	1.2	1.3 (41.0 h)	$1.1 \cdot 10^6$	$4.7 \cdot 10^5$
8	N_2	1.2	1.3 (30.0 h)	182	134
13	N_2	1.3	1.3 (38.4 h)	24	20
18	N_2	1.3	1.4 (41.3 h)	10	9
23	N_2	1.3	1.3 (68.6 h)	6	5
3	*p*-H_2	1.4	1.4 (36.5 h)	$1.1 \cdot 10^6$	$4.7 \cdot 10^5$
3	*p*-H_2	1.4	1.5 (43.8 h)		
3	*p*-H_2	1.4	1.4 (68.8 h)		
3	Ar	1.4	1.5 (39.9 h)		
25	Ar	1.5	1.5 (26.6 h)	5	5
3	Xe	1.3	1.4 (39.2 h)		
35	Xe	1.4	1.4 (38.5 h)	3	3

a: Predicted isotopomeric ratios as derived by Boltzmann distribution using (a) the experimental energy difference $\Delta G(T) = (-\ 0.08 + 0.00007\ T\ K^{-1})\ kcal\ mol^{-1}$,[13] and (b) the calculated ZPVE difference of $\Delta E = -\ 0.08\ kcal\ mol^{-1}$ [B3LYP/6-311G(d,p)] between the isotopomers.

Table S13. Isotopomeric ratio of *CD_3*-**2a** as function of different temperatures and matrix materials.

T / K	Matrix	*CD_3^{open}*-**2a** : *CD_3^{cyc}*-**2a**		predicted ratio (*CD_3*-**2a**)[a]		predicted ratio (*CD_2H*-**2a**)[b]	
		t_0	t_{final}	exp.	calc.	exp.	calc.
3	Ne	1.3	1.4 (16.4 h)	4.9	26.1	2.9	17.2 (sym)
3	Ne	1.2	1.3 (14.1 h)				6.7 (asym)
6	Ne	1.4	1.6 (17.0 h)	2.2	5.1	1.7	4.1 (sym)
6	Ne	1.3	1.4 (17.6 h)				2.6 (asym)

a: Predicted isotopomeric ratios as derived by Boltzmann distribution using (a) the experimental energy difference $\Delta G(T) = (-\ 0.01 + 0.00001\ T\ K^{-1})\ kcal\ mol^{-1}$,[14] and (b) the calculated ZPVE difference of $\Delta E = -\ 0.02\ kcal\ mol^{-1}$ [B3LYP/6-311G(d,p)] between the isotopomers.

b: Predicted isotopomeric ratios as derived by Boltzmann distribution using (a) the experimental energy difference $\Delta G(T) = (-\ 0.006 + 0.00001\ T\ K^{-1})\ kcal\ mol^{-1}$,17 and (b) the calculated ZPVE differences of $\Delta E = -\ 0.017\ kcal\ mol^{-1}$ / $\Delta E = -\ 0.011\ kcal\ mol^{-1}$ [B3LYP/6-311G(d,p)] between the respective symmetric and asymmetric rotamers of the corresponding isotopomers.

Table S14. Isotopomeric ratio of *CD_3*-**2b** as function of different temperatures and matrix materials.

T / K	Matrix	CD_3^{open}-**2b** : CD_3^{cyc}-**2b**		predicted ratio (CD_3-**2b**)[a]	predicted ratio (CD_2H-**2b**)[b]
		t_0	t_{final}	calc.	calc.
3	Ne	0.7	0.9 (94.2 h)	2.9	3.9 (sym)
3	Ne	0.6	0.8 (47.5 h)		1.5 (asym)
6	Ne	0.5	0.8 (50.8 h)	1.7	2.0 (sym)
6	Ne	0.6	0.8 (63.8 h)		1.2 (asym)

a: Predicted isotopomeric ratios as derived by Boltzmann distribution using the calculated ZPVE difference of $\Delta E = -0.006$ kcal mol^{-1} [B3LYP/6-311G(d,p)] between the isotopomers.

b: Predicted isotopomeric ratios as derived by Boltzmann distribution using the calculated ZPVE differences of $\Delta E = -0.008$ kcal mol^{-1} / $\Delta E = -0.003$ kcal mol^{-1} [B3LYP/6-311G(d,p)] between the respective symmetric and asymmetric rotamers of the corresponding isotopomers. (The unphysical inversion of the experimental ratios is attributed to a sligthly erroneous integration in this case due to the low signal-to-noise ratio in the IR spectra.)

Z-Matrices

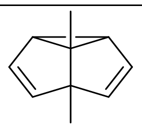

2a

B3LYP/6-311G(d,p)

E = -388.30797551

ZPVE(**2a**) = 0.188334

ZPVE(d^2-**2a**) = 0.185034

ZPVE(d^4-**2a**) = 0.185161

ZPVE(CD_3^{open}-**2a**) = 0.178694

ZPVE(CD_3^{cyc}-**2a**) = 0.178725

	d^2	d^4	CD_3^{open}	CD_3^{cyc}			
C					-0.38646600	0.79118500	0.00044900
C					-1.39389600	0.02775800	-0.80902200
C					-0.68363600	-1.01839100	-1.55419300
C					0.88361600	-0.14658700	-0.00076000
C					0.60139800	-1.08838800	-1.17148100
H					-2.26149100	0.52363300	-1.22987800
H					-1.16256300	-1.67889200	-2.26708700
H					1.32453000	-1.82340100	-1.50736500
C					-1.39257100	0.02732100	0.81097900
H	D				-2.25995400	0.52238900	1.23309000
C					-0.68155300	-1.01997200	1.55392400
H					-1.15924700	-1.68132600	2.26690800
C					0.60312000	-1.08937700	1.16995000
H		D			1.32700800	-1.82411200	1.50475700
C					2.25225300	0.52533700	-0.00064700

	H	D		2.38917400	1.14880100	0.88710800
	H	D		3.04560100	-0.22824100	-0.00148300
	H	D		2.38938100	1.15076800	-0.88698700
	C			-0.27850900	2.29500700	0.00062200
	H		D	0.25115900	2.66052500	0.88576300
	H		D	0.24893300	2.66093100	-0.88572700
	H		D	-1.27506800	2.74556900	0.00197600

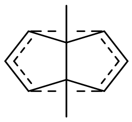	C	-0.79605000	0.38083900	-0.00000200
**TS(2a)**	C	-1.14082100	-0.63706400	-1.04933300
B3LYP/6-311G(d,p)	C	-0.00001800	-1.20682500	-1.59494800
E = -388.30130576	C	0.79606400	0.38081000	-0.00000100
ZPVE(**2a**) = 0.186701	C	1.14079900	-0.63709800	-1.04934100
ZPVE(d_1-**2a**) = 0.183508	H	-2.16231700	-0.86843400	-1.31996200
ZPVE(CD_3-**2a**) = 0.177059	H	-0.00003300	-2.03348100	-2.29466600
	H	2.16228800	-0.86850200	-1.31996700
	C	-1.14082200	-0.63705800	1.04933400
	H	-2.16231900	-0.86842700	1.31996300
	C	-0.00002100	-1.20681600	1.59495300
	H	-0.00003700	-2.03347100	2.29467300
	C	1.14079700	-0.63709200	1.04934500
	H	2.16228600	-0.86849500	1.31997400
	C	1.53775300	1.70691900	-0.00000300
	H	1.29714900	2.29822600	0.88793600
	H	2.61787800	1.53308700	-0.00002000
	H	1.29712300	2.29824000	-0.88792600
	C	-1.53769400	1.70697300	-0.00000400
	H	-1.29706800	2.29827100	0.88793500
	H	-1.29704300	2.29828600	-0.88792600

	H -2.61782400 1.53317900 -0.00002000

		d^2	d^4	CD_3^{open}	CD_3^{cyc}			
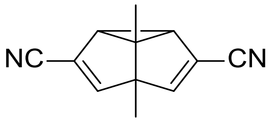	C					0.00000000	1.10984500	0.80547900
	C					-0.79813500	-0.11331600	1.15676600
**2b**	C					-1.53760200	-0.53002500	-0.05111700
B3LYP/6-311G(d,p)	C					-0.00000100	1.12441000	-0.77351300
E = -572.84213729	C					-1.15534700	0.19396200	-1.12539500
ZPVE(**2b**) = 0.186224	H	D				-1.23988200	-0.24691300	2.13642400
ZPVE(d^2-**2b**) = 0.182898	H					-1.50056000	0.03876000	-2.14015800
ZPVE(d^4-**2b**) = 0.183022	C					0.79813800	-0.11331500	1.15676500
ZPVE(CD_3^{open}-**2b**) = 0.176539	H					1.23988700	-0.24691000	2.13642200
	C					1.53760400	-0.53002300	-0.05111900
ZPVE(CD_3^{cyc}-**2b**) = 0.176549	C					1.15534400	0.19396200	-1.12539800
	H		D			1.50055500	0.03876000	-2.14016100
	C					-0.00000200	2.47901800	-1.47102200
	H			D		0.88657600	3.05938500	-1.20504300
	H			D		-0.00000300	2.34932200	-2.55670100
	H			D		-0.88658000	3.05938500	-1.20504100
	C					-0.00000100	2.37583300	1.62365700
	H				D	0.88615300	2.98357400	1.42062700
	H				D	-0.88615700	2.98357200	1.42062800
	H				D	0.00000000	2.13547400	2.68978900
	C					-2.48477900	-1.59052000	-0.06363200
	N					-3.26457300	-2.44359400	-0.04741500
	C					2.48478300	-1.59051500	-0.06363400
	N					3.26457400	-2.44359200	-0.04741600

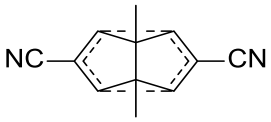 **TS(2b)** B3LYP/6-311G(d,p) E = -572.83245006 ZPVE(**2b**) = 0.186224 ZPVE(*d_1*-**2b**) = 0.181092 ZPVE(*CD_3*-**2b**) = 0.174644	C 0.00000000 1.11507800 0.79467900<br>C -1.01038300 0.06576800 1.14921800<br>C -1.55131600 -0.51410300 0.00000000<br>C 0.00000000 1.11507800 -0.79467900<br>C -1.01038400 0.06576900 -1.14921800<br>H -1.30489800 -0.16110800 2.16356400<br>H -1.30489800 -0.16110800 -2.16356400<br>C 1.01038300 0.06576800 1.14921800<br>H 1.30489800 -0.16110800 2.16356400<br>C 1.55131600 -0.51410400 0.00000000<br>C 1.01038400 0.06576800 -1.14921800<br>H 1.30489800 -0.16110800 -2.16356400<br>C 0.00000000 2.43299300 -1.54613500<br>H 0.88776100 3.02370100 -1.30720800<br>H 0.00000000 2.25255700 -2.62430300<br>H -0.88776000 3.02370100 -1.30720800<br>C 0.00000100 2.43299300 1.54613500<br>H 0.88776100 3.02370100 1.30720800<br>H -0.88776000 3.02370100 1.30720800<br>H 0.00000100 2.25255600 2.62430300<br>C -2.46685400 -1.60561900 0.00000000<br>N -3.22556900 -2.47672100 0.00000000<br>C 2.46685300 -1.60562000 0.00000000<br>N 3.22556800 -2.47672200 0.00000000

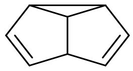 **22** B3LYP/6-311G(d,p)	C 0.00105800 -0.64175700 1.04271500<br>C -0.80409800 -1.18149300 -0.10229300<br>C -1.55329000 -0.05337600 -0.68298300<br>C -0.00066700 0.89707700 0.82062900

E = -309.65191237	C	-1.18164000	1.10984800	-0.12664000
	H	-1.23503500	-2.17497000	-0.07087400
	H	-2.26392900	-0.16131000	-1.49344000
	H	-1.52223100	2.09330100	-0.42820900
	H	0.00178600	-1.08095400	2.03333400
	H	-0.00047100	1.51136500	1.72232200
	C	0.80542000	-1.18095600	-0.10279000
	H	1.23612200	-2.17451100	-0.07230300
	C	1.55272100	-0.05190300	-0.68355300
	H	2.26350300	-0.15788100	-1.49415700
	C	1.18066100	1.11090900	-0.12641300
	H	1.51926100	2.09485700	-0.42871100

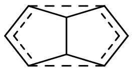 **TS(22)** B3LYP/6-311G(d,p) E = -309.64376586	C	0.00000000	0.78410600	0.90737800
	C	1.05714900	1.14315400	-0.09599200
	C	1.59741100	0.00000000	-0.66806800
	C	0.00000000	-0.78410600	0.90737700
	C	1.05715000	-1.14315400	-0.09599300
	H	1.32629500	2.16316200	-0.33028400
	H	2.29436200	0.00000000	-1.49683500
	H	1.32629500	-2.16316200	-0.33028500
	C	-1.05714900	1.14315400	-0.09599200
	H	-1.32629500	2.16316200	-0.33028400
	C	-1.59741100	0.00000000	-0.66806800
	H	-2.29436200	0.00000000	-1.49683500
	C	-1.05715000	-1.14315400	-0.09599200
	H	-1.32629500	-2.16316200	-0.33028500
	H	0.00000000	-1.28912200	1.87345700
	H	0.00000000	1.28912100	1.87345700

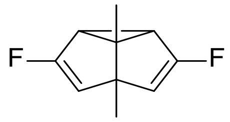

2c

B3LYP/6-311G(d,p)

E = -586.83459191

C	0.00000000	-0.75282100	0.83242900
C	0.80310900	0.47853700	1.12329100
C	1.54149100	0.78903800	-0.10111200
C	0.00000000	-0.84201700	-0.75722100
C	1.19103900	0.03460200	-1.14628400
H	1.24951900	0.66062500	2.09360800
H	1.53919400	0.16034800	-2.16288400
C	-0.80310900	0.47853600	1.12329100
H	-1.24951900	0.66062400	2.09360800
C	-1.54149100	0.78903700	-0.10111200
C	-1.19103900	0.03460100	-1.14628400
H	-1.53919400	0.16034700	-2.16288400
C	0.00000100	-2.24274000	-1.35924300
H	-0.88783000	-2.80434900	-1.05771000
H	0.00000100	-2.18387600	-2.45141800
H	0.88783100	-2.80434900	-1.05771000
C	0.00000100	-1.98164100	1.70482000
H	-0.88562900	-2.59788600	1.52621000
H	0.88563100	-2.59788500	1.52621000
H	0.00000000	-1.69655000	2.76027200
F	2.43541400	1.79623200	-0.11904200
F	-2.43541500	1.79623000	-0.11904200

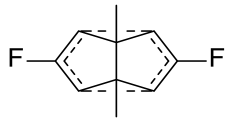

TS(2c)

C	0.00000000	0.76424500	-0.80316000
C	1.06306600	-0.23396400	-1.15312900
C	1.59460800	-0.77546500	-0.00003200
C	0.00000000	0.76418000	0.80322300

B3LYP/6-311G(d,p)	C	1.06306700	-0.23405800	1.15310900
E = -586.82518852	H	1.34700900	-0.48690000	-2.16416900
	H	1.34701000	-0.48707800	2.16412900
	C	-1.06306600	-0.23396600	-1.15312900
	H	-1.34700900	-0.48690300	-2.16416900
	C	-1.59460700	-0.77546700	-0.00003200
	C	-1.06306600	-0.23406000	1.15311000
	H	-1.34700900	-0.48708100	2.16413000
	C	-0.00000200	2.09991600	1.52743900
	H	-0.88832600	2.68634700	1.27880500
	H	-0.00000200	1.93844200	2.60907100
	H	0.88832100	2.68634900	1.27880600
	C	-0.00000200	2.10004100	-1.52726700
	H	-0.88832800	2.68645100	-1.27858700
	H	0.88831900	2.68645600	-1.27858400
	H	0.00000000	1.93865600	-2.60891200
	F	2.48815200	-1.78461800	-0.00007300
	F	-2.48814900	-1.78462200	-0.00007300

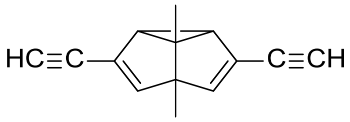	C	0.00000400	1.12610500	0.80768700
	C	-0.79754900	-0.09997100	1.14518900
**2d**	C	-1.54789900	-0.51054400	-0.06344700
B3LYP/6-311G(d,p)	C	-0.00000300	1.15325100	-0.76939300
E = -540.64397953	C	-1.16256800	0.23374600	-1.12559800
	H	-1.23731200	-0.24047400	2.12542400
	H	-1.50332100	0.08809700	-2.14364600
	C	0.79755900	-0.09997200	1.14518300
	H	1.23732900	-0.24047400	2.12541500
	C	1.54789900	-0.51054400	-0.06345900

	C	1.16255900	0.23374500	-1.12560700
	H	1.50330500	0.08809600	-2.14365800
	C	-0.00000500	2.51673600	-1.45086900
	H	0.88709100	3.09497000	-1.17998900
	H	-0.00000900	2.39850200	-2.53829200
	H	-0.88709800	3.09497000	-1.17998200
	C	0.00000700	2.38488300	1.63711300
	H	0.88594600	2.99579500	1.44008100
	H	-0.88593300	2.99579500	1.44008800
	H	0.00001100	2.13504700	2.70145200
	C	-2.48896200	-1.57134000	-0.07273800
	C	2.48896100	-1.57134000	-0.07275800
	C	-3.29512500	-2.46676400	-0.04440800
	H	-4.00266200	-3.25883000	-0.03091500
	C	3.29512300	-2.46676600	-0.04442500
	H	4.00264300	-3.25884500	-0.03079900

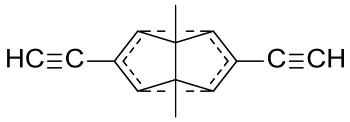 **TS(2d)** B3LYP/6-311G(d,p) E = -540.63352738	C	0.00000000	1.13257100	-0.79368600
	C	1.01887600	0.09163100	-1.14499500
	C	1.56753000	-0.49478800	-0.00001100
	C	0.00000000	1.13253600	0.79373600
	C	1.01887600	0.09158000	1.14499900
	H	1.30558000	-0.13772100	-2.16138100
	H	1.30557900	-0.13781900	2.16137500
	C	-1.01887600	0.09163100	-1.14499500
	H	-1.30558000	-0.13772100	-2.16138100
	C	-1.56753000	-0.49478800	-0.00001100
	C	-1.01887600	0.09158000	1.14499900
	H	-1.30557900	-0.13781900	2.16137500

	C	0.00000000	2.45346500	1.54157300
	H	-0.88798500	3.04461900	1.30236900
	H	0.00000000	2.27493100	2.62043400
	H	0.88798500	3.04461900	1.30236900
	C	0.00000000	2.45353400	-1.54146300
	H	-0.88798500	3.04467700	-1.30223400
	H	0.88798500	3.04467700	-1.30223400
	H	0.00000000	2.27504700	-2.62033300
	C	2.48353500	-1.58256100	-0.00003500
	C	-2.48353500	-1.58256100	-0.00003500
	C	3.27457300	-2.49066200	-0.00005500
	H	3.96669300	-3.29626800	-0.00007200
	C	-3.27457300	-2.49066200	-0.00005500
	H	-3.96669300	-3.29626800	-0.00007200

Literature references

[1] a) Y. P. Liu, G. C. Lynch, T. N. Truong, D. H. Lu, D. G. Truhlar and B. C. Garrett, J. Am. Chem. Soc. 1993, 115, 2408-2415; b) D.-h. Lu, T. N. Truong, V. S. Melissas, G. C. Lynch, Y.-P. Liu, B. C. Garrett, R. Steckler, A. D. Isaacson, S. N. Rai, G. C. Hancock, J. G. Lauderdale, T. Joseph and D. G. Truhlar, Comput. Phys. Commun. 1992, 71, 235-262.

[2] J. Zheng, J. L. Bao, R. Meana-Pañeda, S. Zhang, B. J. Lynch, J. C. Corchado, Y.-Y. Chuang, P. L. Fast, W.-P. Hu, Y.-P. Liu, G. C. Lynch, K. A. Nguyen, C. F. Jackels, A. F. Ramos, B. A. Ellingson, V. S. Melissas, J. Villà, I. Rossi, E. L. Coitiño, T. V. A. J. Pu, A. Ratkiewicz, R. Steckler, B. C. Garrett, A. D. Isaacson and D. G. Truhlar in POLYRATE Version 2016-2A, Vol. University of Minnesota, Minneapolis, 2016.

[3] J. Zheng, J. L. Bao, S. Zhang, J. C. Corchado, R. Meana-Pañeda, Y.-Y. Chuang, E. L. Coitiño, B. A. Ellingson and D. G. Truhlar in GAUSSRATE 17, Vol. University of Minnesota, Minneapolis, 2017.

[4] M. J. Frisch, G. W. Trucks, H. B. Schlegel, G. E. Scuseria, M. A. Robb, J. R. Cheeseman, G. Scalmani, V. Barone, B. Mennucci, G. A. Petersson, H. Nakatsuji, M. Caricato, X. Li, H. P. Hratchian, A. F. Izmaylov, J. Bloino, G. Zheng, J. L. Sonnenberg, M. Hada, M. Ehara, K. Toyota, R. Fukuda, J. Hasegawa, M. Ishida, T. Nakajima, Y. Honda, O. Kitao, H. Nakai, T. Vreven, J. A. Montgomery Jr., J. E. Peralta, F. Ogliaro, M. J. Bearpark, J. Heyd, E. N. Brothers, K. N. Kudin, V. N. Staroverov, R. Kobayashi, J. Normand, K. Raghavachari, A. P. Rendell, J. C. Burant, S. S. Iyengar, J. Tomasi, M. Cossi, N. Rega, N. J. Millam, M. Klene, J. E. Knox, J. B. Cross, V. Bakken, C. Adamo, J. Jaramillo, R. Gomperts, R. E. Stratmann, O. Yazyev, A. J. Austin, R. Cammi, C. Pomelli, J. W. Ochterski, R. L. Martin, K. Morokuma, V. G. Zakrzewski, G. A. Voth, P. Salvador, J. J. Dannenberg, S. Dapprich, A. D. Daniels, Ö. Farkas, J. B. Foresman, J. V. Ortiz, J. Cioslowski and D. J. Fox in Gaussian 09, Vol. Gaussian, Inc., Wallingford, CT, USA, 2009.

[5] a) A. D. Becke, J. Chem. Phys. 1993, 98, 5648-5652; b) B. Miehlich, A. Savin, H. Stoll and H. Preuss, Chem. Phys. Lett. 1989, 157, 200-206; c) C. Lee, W. Yang and R. G. Parr, Phys. Rev. B 1988, 37, 785-789.

[6] P. C. Hariharan and J. A. Pople, Theor. Chim. Acta 1973, 28, 213-222.

[7] X. Zhang, D. A. Hrovat and W. T. Borden, Org. Lett. 2010, 12, 2798-2801.

[8] S. Grimme, J. Antony, S. Ehrlich and H. Krieg, J. Chem. Phys. 2010, 132, 154104.

[9] M. Page and J. W. McIver, J. Chem. Phys. 1988, 88, 922-935.

[10] S. Pierau in Neue Methoden zur Diastereo- und Enantioselektiven Synthese von 8-oxabicyclo[3.2.1]oct-6-en-3-onen, Vol. PhD Universität Hannover, Hannover, 1997.

[11] M. Zhan, T. Zhang, H. Huang, Y. Xie and Y. Chen, J. Label. Compd. Radiopharm. 2014, 57, 533-539.

[12] K. Abbaspour Tehrani, M. Boeykens, V. I. Tyvorskii, O. Kulinkovich and N. De Kimpe, Tetrahedron 2000, 56, 6541-6548.

[13] H. Quast, Y. Görlach, E.-M. Peters, K. Peters, H. G. von Schnering, L. M. Jackman, G. Ibar and A. J. Freyer, Chem. Ber. 1986, 119, 1801-1835.

[14] R. Askani, H.-O. Kalinowski, B. Pelech and B. Weuste, Tetrahedron Lett. 1984, 25, 2321-2324.
